# Cell Death Mechanism of Organometallic Ruthenium(II) and Iridium(III) Arene Complexes on HepG2 and Vero Cells

**DOI:** 10.1021/acsomega.3c05898

**Published:** 2023-09-27

**Authors:** Serdar Batıkan Kavukcu, Hilal Kabadayı Ensarioğlu, Hande Karabıyık, Hafize Seda Vatansever, Hayati Türkmen

**Affiliations:** † Ege University, Faculty of Science, Department of Chemistry, Izmir 35100, Turkey; ‡ Manisa Celal Bayar University, Faculty of Medicine, Department of Histology and Embryology, Manisa 45030, Turkey; § Dokuz Eylül University, Faculty of Science, Department of Physics, Izmir 35390, Turkey; ∥ Near East University, DESAM Institute, Mersin 10, Turkey 99138

## Abstract

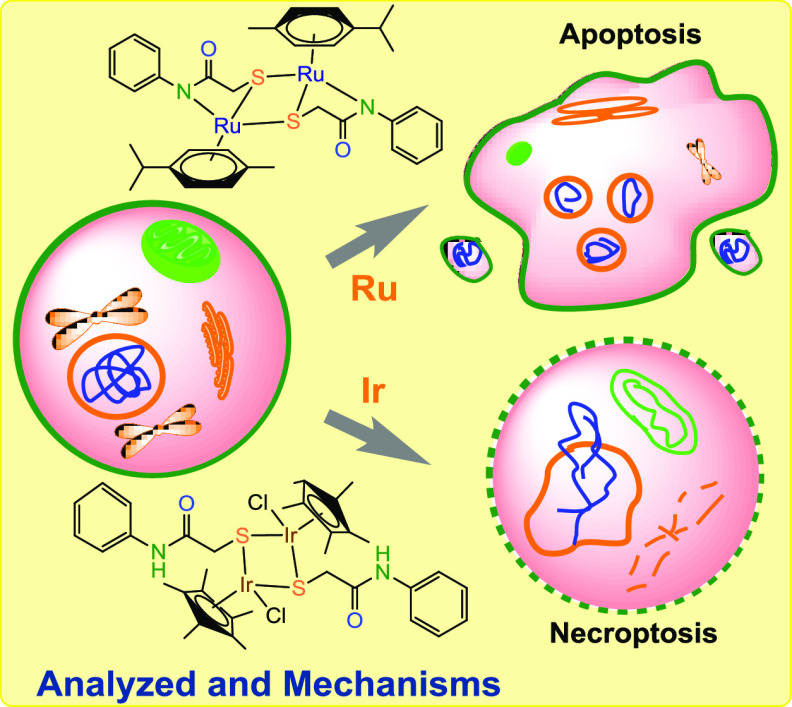

Due to side effects and toxicity associated with platinum-derived metal-based drugs, extensive research has been conducted on ruthenium (Ru) complexes. We aim to synthesize a highly oil soluble Ru(II)–*p*-cymene complex (**Ru1**) with an aliphatic chain group, a bimetallic Ru(II)–*p*-cymene complex (**Ru2**) with N,S,S triple-coordination and a bimetallic Ir(III)–pentamethylcyclopentadienyl complex (**Ir1**) with S,S double-coordination. Subsequently, we investigate the effects of these complexes on Vero and HepG2 cell lines, focusing on cell death mechanisms. Characterization of the complexes is performed through nuclear magnetic resonance spectroscopy (^1^H and ^13^C NMR) and Fourier-transform infrared spectroscopy. The effective doses are determined using the (3-(4,5-dimethylthiazol-2-yl)-2,5-diphenyl-2*H*-tetrazolium bromide) (MTT) assay, applying different doses of the complexes to the two cell lines for 24 and 48 h, respectively. Immunoreactivities of Bax, Bcl2, caspase-3, RIP3, and RIPK1 are analyzed using the indirect immunoperoxidase technique. Notably, all the complexes (**Ru1**, **Ru2**, and **Ir1**) exhibit distinct cell death mechanisms, showing greater effectiveness than cisplatin. This study reveals the diverse mechanisms of action of Ru and Ir complexes based on different ligands. To the best of our knowledge, this study represents the first investigation of a novel RAED-type complex (**Ru1**) and unexpected bimetallic complexes (**Ru2** and **Ir1**).

## 1 Introduction

Liver malignancy is a serious global health concern, with increasing incidence worldwide. Hepatocellular carcinoma, which originates from hepatocytes, is the most common histological subtype, constituting 75–90% of the total liver cancer cases diagnosed globally.^1−3^ Novel metal-based chemotherapeutics are being increasingly studied by the research community owing to their high activity and low toxicity.^4−6^ Since Rosenborg’s discovery in 1965, cisplatin and its derivatives are still widely used in cancer treatment.^7−9^ However, owing to the limitations of cisplatin, such as toxicity, drug resistance, and nonselectivity, developing anticancer drugs based on other metals, particularly platinum group metals, became necessary.^10a,11^ Accordingly, ruthenium (Ru), a platinum group metal, gained prominence because it exhibited remarkable results.^12^ The ruthenium age began with the Ru(III) complex *fac*-[RuCl_3_(NH_3_)_3_], which was reported by Clarke.^13^ Other Ru(III) complexes, such as NAMI-A and K1019, exhibited effects in metastatic cancers and primary tumors, respectively.^14,15^ Ru is present in oxidation steps II, III, and IV in its complexes. Ru complexes are considered less toxic than platinum complexes owing to their mechanism of transport into the cell.^16^ Ru complexes mimic iron and are transported by binding to serum transferrin and albumin biomolecules in the blood, making them selective for cancer cells.^17,18^ The cytotoxic properties of half-sandwich, piano-stool Ru(II) complexes were discovered by Sadler et al.^19^ Ru(II)–arene complexes are basically divided into two classes: RAED ([Ru(η^6^-arene)(en)Cl]^+^ (en: ethylenediamine)) and RAPTA ([Ru(η^6^-arene) (PTA)X_6_] (PTA: 1,3,5-triaza-7-phosphoadamantane)).^16,20,21^ Promising results were obtained in treating human ovarian cancer cells using RAED complexes. The general formula of organo-Ru(II) half-sandwich complexes is [(η^6^-arene)Ru(X)(L)], where X represents a halogen atom while L can be a single- or double-bonded ligand. Cyclometalated iridium(III) complexes have received extensive research attention in recent years owing to their potential anticancer properties.^22^ These complexes are known to possess remarkably high stability and low toxicity,^23^ making them attractive candidates for the development of new cancer treatments.^24^

The layout and structure of a ligand are critical in deciding the pharmacological properties of complexes.^25^ Moreover, molecular weight, hydrogen donor and acceptor abilities, and active site binding need to be considered. However, the biggest problem with complexes that contain two or more metals is the decrease in uptake into the cell with increasing molecular weight. Therefore, we must prepare polymetallic systems with as small ligand systems as possible. Particularly, dimeric, bimetallic complexes can interact more with a target by breaking the weak bridging bonds in solution, creating coordination gaps in the metal center. Thus, with the appropriate choice of ligands, the effectiveness and cytotoxicity of a complex increase considerably.^26^

Programmed cell death (PCD) is critical in the development, homeostasis, control, and progression of many diseases, including cancer and neurodegenerative pathologies. Besides classical apoptosis, several different forms of PCD have now been recognized, including necroptosis. Apoptosis is an evolutionarily conserved process that is morphologically characterized by various features, including pyknosis, membrane blebbing, nuclear fragmentation, nuclear condensation, and apoptotic body formation. Necroptosis is a form of PCD that, similar to extrinsic apoptosis, can be executed upon activation of a death receptor, as well as some Toll-like receptors and the intracellular sensor DAI/ZBP1. Necroptosis is morphologically and mechanistically distinct from apoptosis. Extrinsic apoptosis depends on initiator- and effector-caspase activation, whereas necroptosis is a kinase-mediated pathway.^27^

We report the synthesis and characterization of novel Ru(II)– and Ir(III)–arene complexes with different aromatic and aliphatic groups and then investigate their effects on Vero and HepG2 cell lines through cell death mechanisms. Moreover, we compare the N–N and N–S donor systems. We prepare two different metal complexes based on the N–S donor ligand and study their behaviors with respect to the metal center and investigate their cell death mechanisms via immunohistochemical analysis. Overall, Ru and Ir complexes hold extensive promise as potential anticancer drugs. Further studies are needed to completely understand their mechanisms of action and optimize their therapeutic potential. However, the unique properties of Ru and Ir complexes make them an exciting candidate for development of new cancer treatments.

## 2 Results and Discussion

### 2.1 Chemistry

**Scheme 1:**
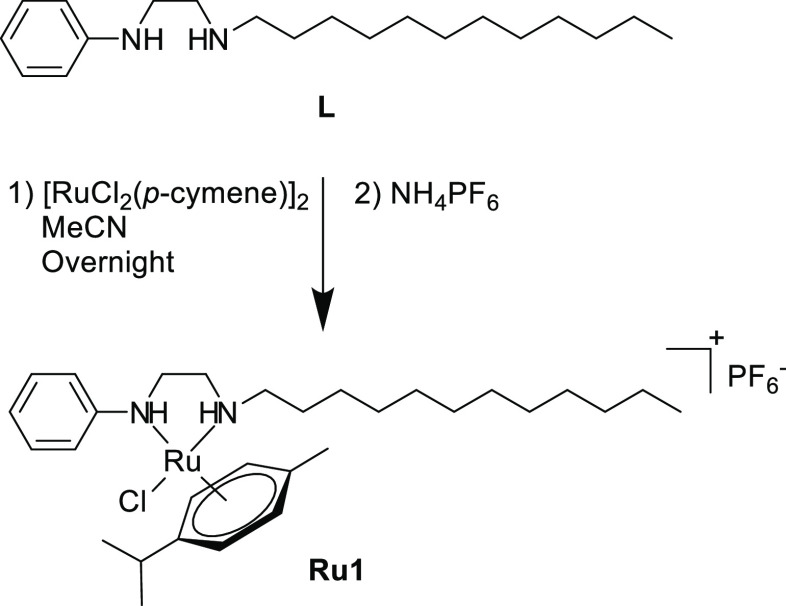
Synthesis of **Ru1**

Recently, the cytotoxic properties of mono- and bimetallic Ru(II)–*p*-cymene complexes containing N,N donor ethylenediamine in a wide range of cell lines were investigated, and promising results were obtained.^10a^ This study led us to examine different structures of Ru(II)–arene complexes. Accordingly, we prepared an N,N-coordinated, cationic Ru(II)–*p*-cymene complex (**Ru1**), which can pass through the cell wall owing to its satisfactory solubility, an N,S,S-coordinated, bimetallic Ru(II)–*p*-cymene complex (**Ru2**), and an S,S-coordinated, bimetallic Ir(III)–pentamethylcyclopentadienyl complex (**Ir1**). Scheme 1 shows the synthesis method of **Ru1**, which was prepared according to our published procedure.^10b^**Ru1** was an air- and moisture-stable orange solid and well soluble in apolar solvents. It exhibited a cationic structure that formed a chelate complex. The stability of **Ru1** was ensured by the PF_6_^–^ anion.

**Scheme 2:**
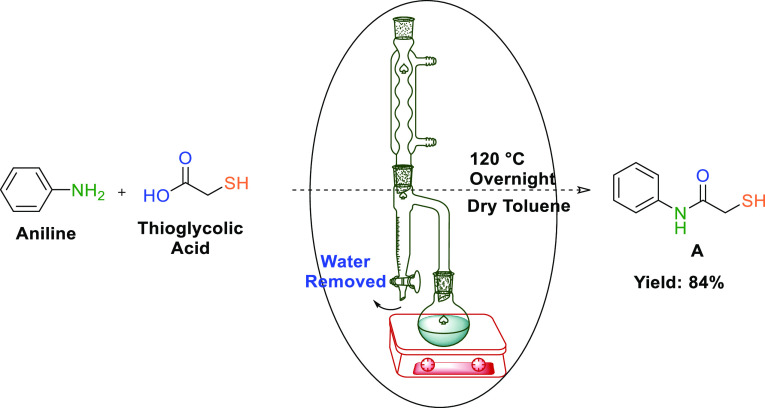
Synthesis of Ligand **A**

Scheme 2 shows the synthesis pathway of **A**. Ligand **A** was prepared via the reaction between aniline and thioglycolic acid through refluxing in dry toluene using a Dean–Stark apparatus. This reaction is a condensation reaction during which water is released. The Dean–Stark apparatus is essential for the formation of the ligand. Notably, the water in the solution must be removed for the reaction equilibrium to shift toward the products.

**Scheme 3:**
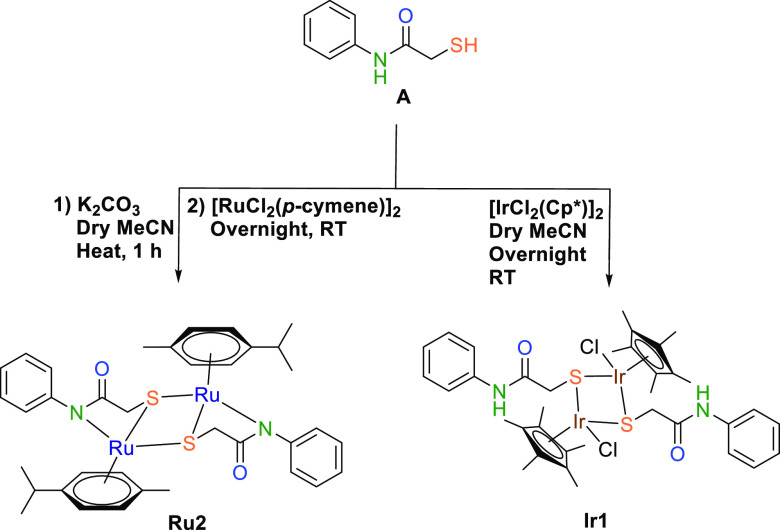
Synthesis of Complexes **Ru2** and **Ir1**

Scheme 3 illustrates the synthesis pathways of **Ru2** and **Ir1**. The Ru complex (**Ru1**) of ligand **A** was synthesized using [RuCl_2_(*p*-cymene)]_2_, K_2_CO_3_, and dry MeCN. Surprisingly, Ru exhibited a tricoordinated, dimeric, bimetallic structure, forming a neutral complex. Consequently, an unexpected structure with N,S,S coordination was obtained. Attempting the reaction without potassium carbonate did not yield a stable complex. In other words, a bidentate complex with S,S donors, like **Ir1**, was not observed for the ruthenium complex. This discrepancy may be attributed to charge equivalence. When a proton is removed from the amide nitrogen, that nitrogen becomes minus charged. As a result, ruthenium, with an oxidation state of +2, formed a tridentate complex to achieve charge balancing with the negatively charged nitrogen. The iridium (Ir) complex (**Ir1**) of ligand **A** was prepared using [IrCl_2_(Cp*)]_2_ in dry MeCN at room temperature. Interestingly, Ir1 also formed a bimetallic, dimeric, neutral complex, characterized by a thio-bridged, S,S-coordinated structure. Since the Cp* ring carries a negative charge, **Ir1** did not require nitrogen coordination. **Ru2** and **Ir1** were characterized through ^1^H and ^13^C nuclear magnetic resonance (NMR) spectroscopy, Fourier-transform infrared spectroscopy (FTIR), and single-crystal X-ray diffraction (XRD) techniques. Additionally, they were analyzed using 2D ^1^H–^1^H gHSQC and ^1^H–^13^C gCOSY NMR spectroscopies. The purity of the complexes was determined by elemental analysis before the biological testing. The complexes were obtained with satisfactory yields and were air- and moisture-stable in the form of yellow–orange solids.

The formation of the complexes was supported by evaluating the chemical shifts and splits in the assignment of the ^1^H NMR resonances of the complexes. The aromatic protons of the phenyl ring were shifted to 7.41 and 7.18 ppm as a multiplet for **Ru2**. The C*H*_2_ protons were split as a quartet with a high *J* value at 3.56–3.36 ppm. Normally, these CH_2_ aliphatic protons were observed as a singlet in the ^1^H NMR spectrum of **A**. This framework-type quartet cleavage is evidence that the chemical environment of these protons had changed. The aromatic *p*-cymene-C*H* protons were divided into four different doublets, each with one proton. This is an indication of the formation of a chelate complex, similar to that in our previous studies.^10^ Other *p*-cymene aliphatic protons were observed in the low ppm/high area, as expected. The aromatic protons of the phenyl ring were shifted to 7.63, 7.33, and 7.18 ppm as doublet, triplet, and triplet for **Ir1**. The C*H*_2_ protons were observed at 3.98 ppm as a singlet for **Ir1**. The methyl protons of pentamethylcyclopentadienyl were detected as a sole singlet as expected.

The FTIR analysis results of the complexes are presented in the Supporting Information (Figures S6–S8). Normally, a weak stretching peak appeared between 2550 and 2600 cm^–1^ corresponding to the thiol bond (S–H).^28^ This peak, however, did not appear in either complex (**Ru2** or **Ir1**), confirming that the proton of the S–H bond is detached during metal coordination. One of the most important differences between the two bimetallic complexes (**Ru2** and **Ir1**) is that Ru is coordinated from the nitrogen and the proton of the nitrogen is separated, whereas the amide group remains in the Ir complex. In **Ir1**, the presence of a considerably strong absorption frequency at 1679 cm^–1^ is related to the amide functional group.^29^ The equivalent of this strong absorption frequency peak in **Ru2** is at 1537 cm^–1^. Similarly, in **Ir1**, the peak observed at 3493 cm^–1^ corresponds to N–H stretching; however, this stretching was not observed in **Ru2**. The complexes showed bands, 2900–3200 and 750–800 cm^–1^ regions, which were assumed to be indicative of the C–H vibrations. The C–N and C=C vibrations were observed in the regions of 1000–1250 and 1600 cm^–1^, respectively. Additionally, metal–halogen vibrations can be seen in the fingerprint area.^10^

**Figure 1:**
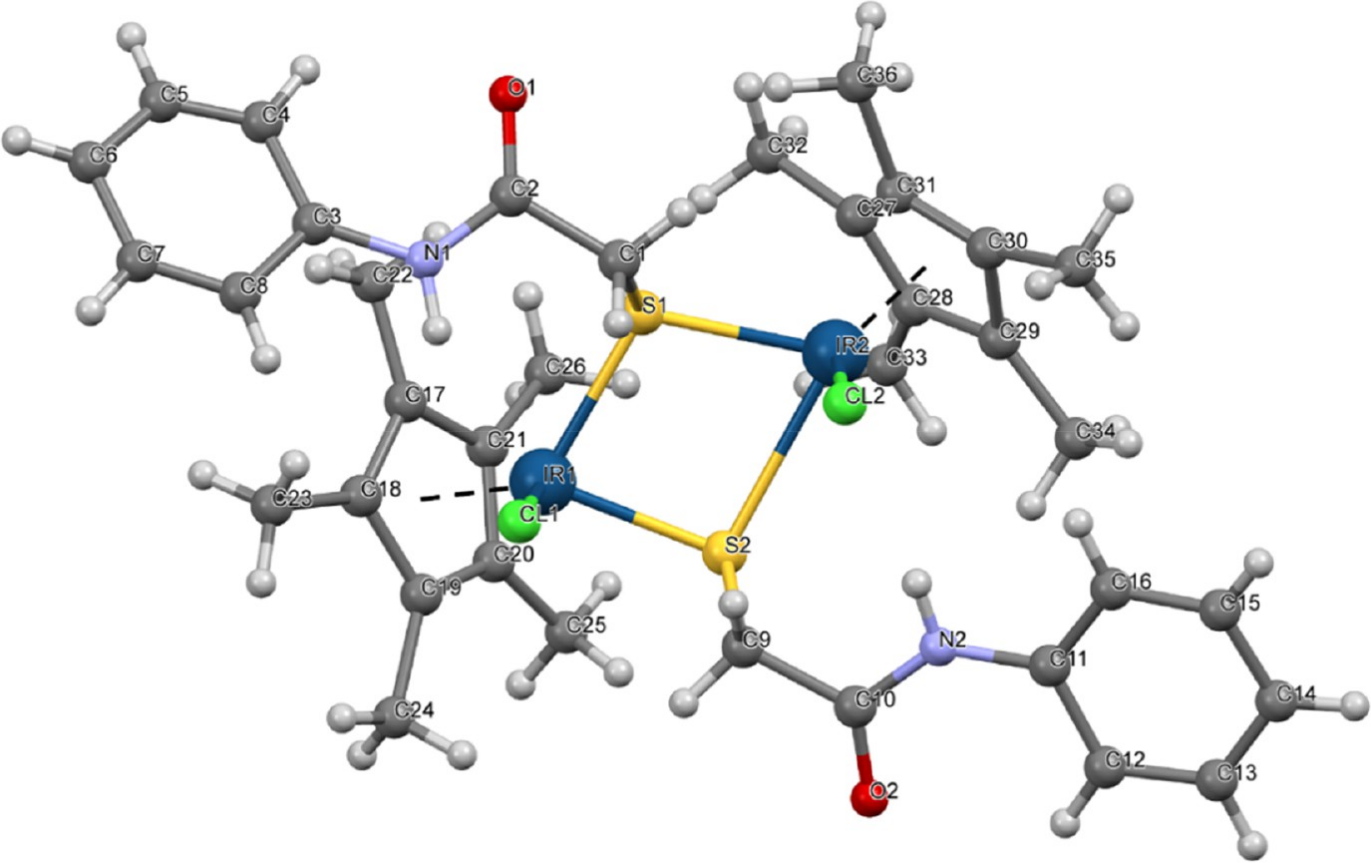
Molecular structure of **Ir1** showing the atom numbering scheme.

**Figure 2:**
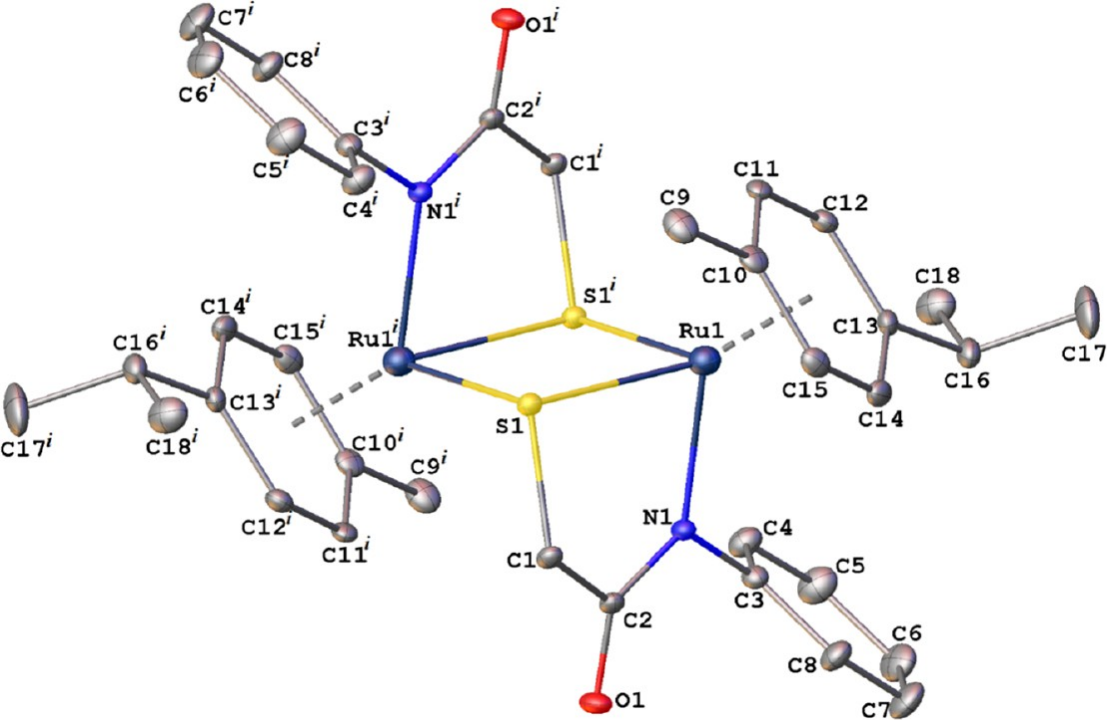
Molecular structure of **Ru2** with thermal ellipsoids plotted at the 30% probability level. Symmetry-related atoms are labeled with superscript *i*. Symmetry code: (i) 1 – *x*, 1 – *y*, 1 – *z*. Hydrogen atoms are omitted for clarity.

Single crystals for the solid-state structures were obtained via the diffusion of diethyl ether into concentrated solutions of the complexes in dichloromethane. **Ru2** and **Ir1** exhibited a piano-stool geometry. Figure 1 shows the molecular structure of **Ir1**, along with the atom numbering scheme. The asymmetric unit of **Ir1** contains two Ir(III) ions, two **A** ligands, two pentamethylcyclopentadienyl anions, and two coordinated chloride ligands. The bond distances of Ir–S are between 2.330(8) and 2.761(16) Å and those of Ir–Cl are between 2.407(5) and 2.410(5) Å. Table 1 presents selected bond distances and angles for **Ir1**. The asymmetric unit of **Ru2** (Figure 2) contains a half molecule, whereas the entire molecule is complemented by inversion symmetry with respect to the center of mass of the Ru1–S1–Ru1i–S1i parallelogram. Ru1–S1 and Ru1–S1i bond distances are 2.372(1) and 2.389(1) Å, respectively. The distance between the Ru atom and the centroid of the *p*-cymene ring is 1.714(2) Å, and the Ru–C bond distances are in the range of 2.197(5)–2.264(5) Å. Table 2 presents selected bond distances and angles with respect to **Ru2**.

**Table 1:** Selected Bond Distances and Angles with Respect to **Ir1** (Å, deg)

Ir1–Cl1	2.410 (5)	Ir2–Cl2	2.407 (5)
Ir1–S1	2.379 (6)	Ir1–S2	2.330 (8)
Ir2–S1	2.363 (7)	Ir2–S2	2.761 (16)
S2–Ir1–S1	81.7 (4)	S2–Ir1–Cl1	95.2 (3)
S1–Ir1–Cl1	92.48 (19)	S1–Ir2–S2	73.5 (2)
S1–Ir2–Cl2	92.6 (2)	S2–Ir2–Cl2	87.8 (2)

**Table 2:** Selected Bond Distances and Angles with Respect to **Ru2** (Å, deg)

Ru1–S1	2.372 (1)	Ru1–S1^*i*^	2.389(1)
Ru1–N1	2.130(4)	S1–C1	1.820(5)
C2–C1	1.522(6)	N1–C2	1.344(6)
O1–C2	1.241(6)	N1–C3	1.442(6)
N1–Ru1–S1	80.78(11)	N1–Ru1–S1^*i*^	83.29(11)
C1–S1–Ru1	96.72(16)	C1–S1–Ru1^*i*^	108.61(17)
S1–Ru1–S1^*i*^	79.06(4)	C2–N1–Ru1	122.1(3)

**Figure 3:**
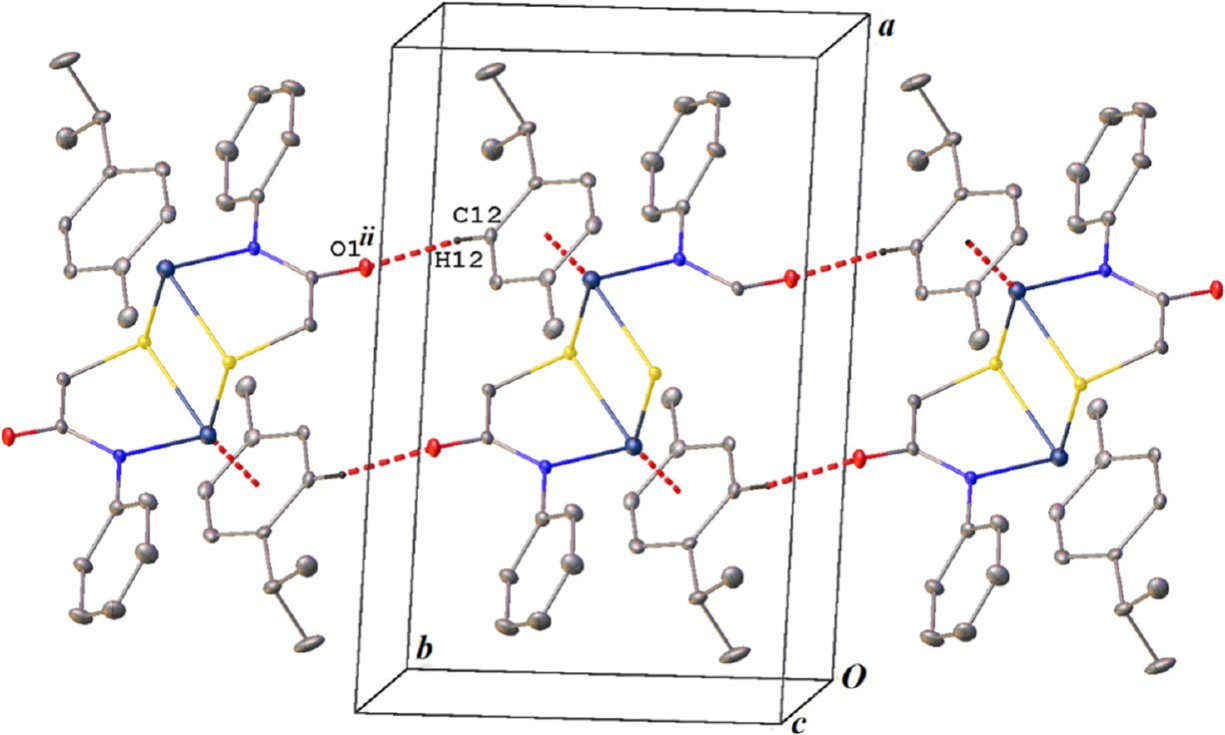
Intermolecular C―H---O hydrogen bonds along the *b*-axis of the unit cell. Symmetry code: (ii) *x*, 1 + *y*, *z*. Hydrogen atoms not involved in the interactions have been omitted for clarity.

A weak intermolecular C12–H12---O1 hydrogen bond exists in the crystal structure of **Ru2** (Figure 2). H12---O1 and C12---O1 distances are 2.351(1) and 3.271(1) Å, respectively, and the C12–H12---O1 angle is 169.9(1)°. Hydrogen-bonded molecules are aligned along the *b*-axis of the unit cell (Figure 3).

### 2.2 Pharmacology/Biology

#### 2.2.1 Cell Viability and Cytotoxicity

**Figure 4:**
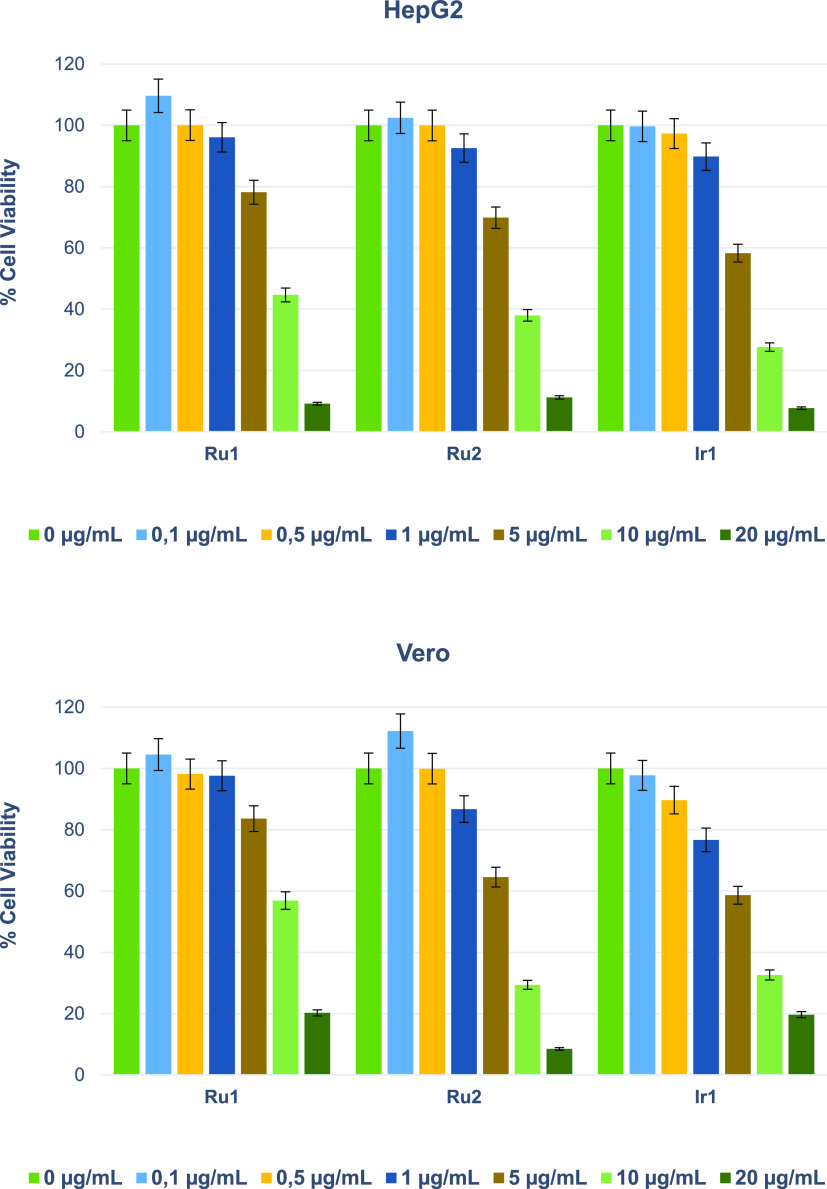
Cell viability results (mean ± standard deviation) of the complexes (**Ru1–2** and **Ir1**) in HepG2 and Vero cell lines after 24 h of exposure.

HepG2 and Vero cells were treated with different concentrations of **Ru1, Ru2,** and **Ir1** complexes for 24 and 48 h. Cell viability was determined using the (3-(4,5-dimethylthiazol-2-yl)-2,5-diphenyl-2*H*-tetrazolium bromide) (MTT) assay. Figure 4 shows the cell viability values of **Ru1**, **Ru2**, and **Ir1**.  Our results showed that for a 24-h incubation period, **Ru1, Ru2**, and **Ir1** inhibited HepG2 and Vero cell growth more effectively at 10 and 20 μg/mL concentrations (Figure 4). The 11.10 μM concentration was determined to be the ideal concentration for cell morphology and immunohistochemical analysis for all complexes. We could have opted for a higher concentration in our subsequent studies. However, our decision to use the current concentration was driven by the desire to avoid excessive lethality and toxicity to the cells, thus enabling us to observe cell changes more accurately. Furthermore, it is important to note that both **Ru2** and **Ir1** complexes contain two metal centers in their structure, and their dimeric nature causes them to behave as two separate complexes in solution (Figure 13). As a result, the concentration we actually worked with can be considered double the nominal concentration. Therefore, we conducted our experiments at an effective concentration of 11.10 μM, a choice that provided us with results conducive to a more in-depth analysis of the complexes’ mechanisms.

#### 2.2.2 Cell Morphology

**Figure 5:**
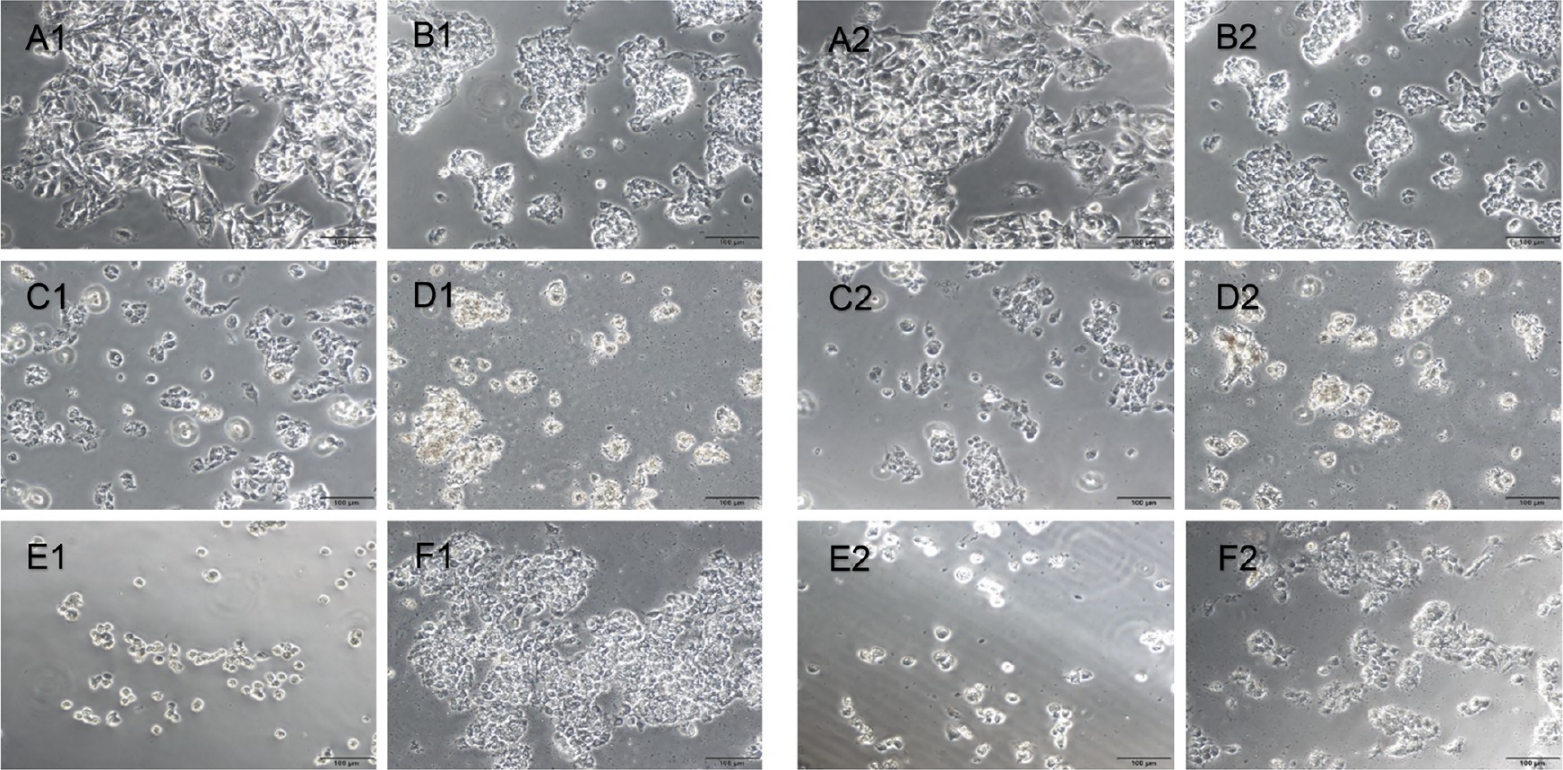
HepG2 cell culture in control (A), DMSO (B), cisplatin (C), **Ru1** applied (D), **Ru2** applied (E), and **Ir1** applied (F) groups after 24 h (left) and 48 h (right) of incubation. Scale bars: 100 μm.

**Figure 6:**
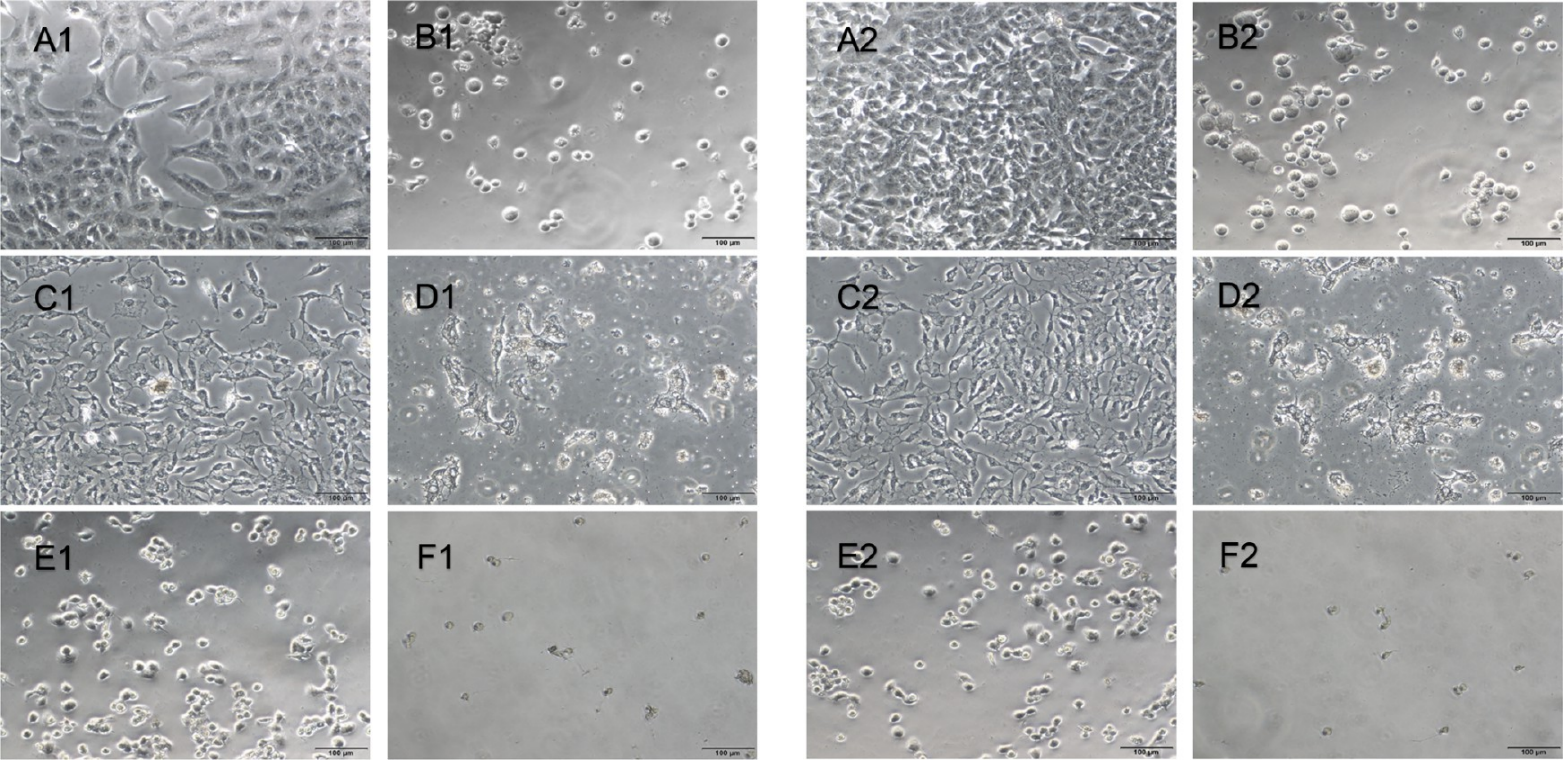
Vero cell culture in control (A), DMSO (B), cisplatin (C), **Ru1** applied (D), **Ru2** applied (E), and **Ir1** applied (F) groups after 24 h (left) and 48 h (right) of incubation. Scale bars: 100 μm.

HepG2 cells exhibited an epithelioid-like structure (Figures 5A1 and 5A2), and Vero cells displayed a fibroblastic feature after 24 and 48 h (Figures 5 and 6) in culture. After treatment with the **Ru1** (Figures 5D1, 5D2, 6D1, and 6D2), **Ru2** (Figures 5E1, 5E2, 6E1, and 6E2), and **Ir1** (Figures 5F1, 5F2, 6F1, and 6F2) complexes, the shape of the cells changed to oval and their number decreased in the HepG2 (Figure 5) and Vero (Figure 6) cell lines at incubation for 24 and 48 h. Fewer cells were observed upon cisplatin application in HepG2 (Figure 5) and Vero (Figure 6) cells compared with application of the **Ru2** (Figures 5E1, 5E2, 6E1, and 6E2) and **Ir1** (Figures 5F1, 5F2, 6F1, and 6F2) complexes. However, a similar number of cells were detected when cisplatin and **Ru1** (Figures 5D1, 5D2, 6D1, and 6D2) were applied to both type of cells. While similar morphology and number of HepG2 cells were detected after 24- and 48-h application with **Ru1** and **Ru2**, respectively, the number of HepG2 cells was less after **Ir1** application at 48 h (Figure 5F2) than at 24 h (Figure 5F1). Larger versions of the images are in the Supporting Information (Figures S14–S17).

#### 2.2.3 Immunohistochemical Evaluation

**Figure 7:**
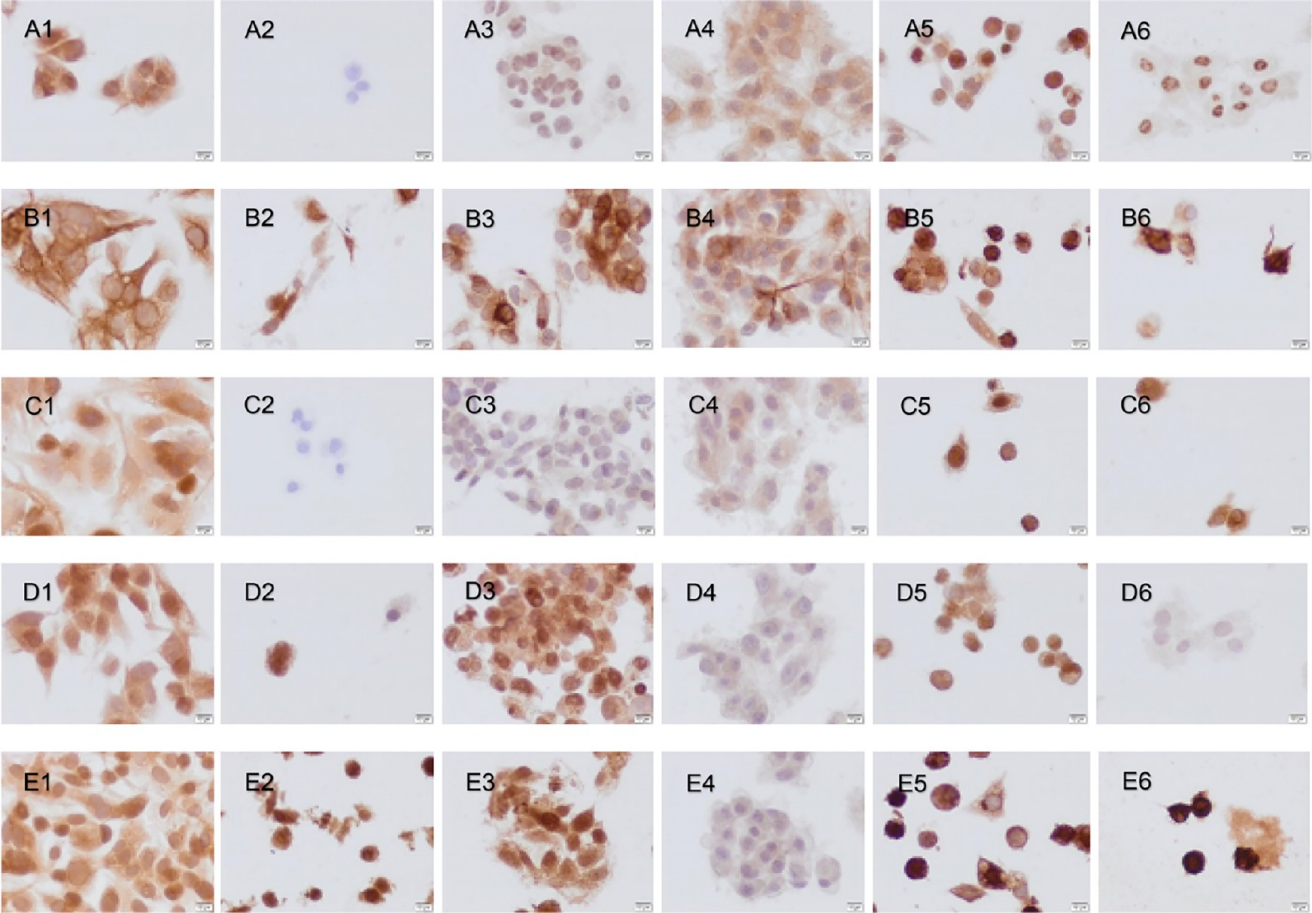
Bax (A1–A6), Bcl2 (B1–B6) Cas3 (C1–C6), RIP3 (D1–D6), and RIPK1 (E1–E6) immunoreactivities after control (A1–E1), DMSO (A2–E2), cisplatin (A3–E3), **Ru1** (A4–E4), **Ru2** (A5–E5), and **Ir1** (A6–E6) administration to HepG2 cells. Scale bars: 10 μm.

**Figure 8:**
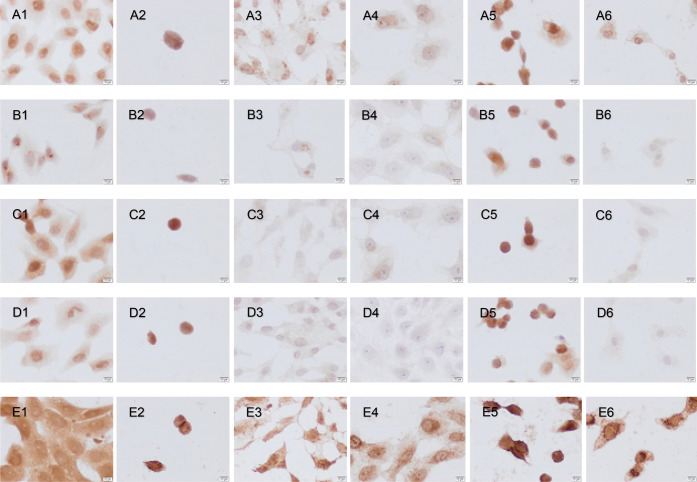
Bax (A1–6), Bcl2 (B1–6) Cas3 (C1–6), RIP3 (D1–6), and RIPK1 (E1–6) immunoreactivities after control (A1–E1), DMSO (A2–E2), cisplatin (A3–E3), **Ru1** (A4–E4), **Ru2** (A5–E5), and **Ir1** (A6–E6) administration to Vero cells. Scale bars: 10 μm.

Immunohistochemistry is used to study the distribution and localization of specific proteins or biomolecules within cancer cells or tumor tissues. Immunohistochemistry provides insight into Ru(II) arene complexes’ mechanism of action and potential therapeutic applications. We performed the immunohistochemical analysis with Bax, Bcl2, caspase 3, RIP3, and RIPK1 on HepG2 and Vero cells to determine the cell death mechanism. In control HepG2 cells, low immunoreactivities of Bax, Bcl2, caspase-3, RIP3, and RIPK1 were detected (Figure 7). After dimethyl sulfoxide (DMSO) (Figures 7A2–E2) and cisplatin (Figures 7A3–E3) application, Bax and caspase-3 immunoreactivities were negative, whereas bcl-2, RIP3, and RIPK1 immunoreactivities were detected on HepG2 cells. In Vero cells, caspase-3 and RIPK1 immunoreactivities were more detectable after cisplatin application (Figures 8A3–E3). After **Ru1** administration, weak Bax, Bcl2, and caspase-3 immunoreactivities were detected in HepG2 cells (Figures 7A4–E4) and negative immunoreactivities in Vero cells (Figures 8A4–E4). Caspase-3, RIP,3 and RIPK1 immunoreactivities were observed to be moderate in both cell lines after **Ru2** application (Figures 7A5–E5, Figures 8A5–E5) and similar to that for cisplatin administration in HepG2 cells (Figures 7A3–E3). After **Ir1** administration, an increase in RIPK1 immunoreactivity was primarily detected (Figures 7 and 8A6–E6). Larger versions of the images are in the Supporting Information (Figures S18–S29).

We observed that **Ru1** activated apoptosis, particularly in HepG2 cells, and **Ru2** and **Ir1** had an effect on activating necroptosis by increasing RIPK1 immunoreactivity in both cell lines. The N–N-donor Ru(II) complex (**Ru1**) activated the apoptosis mechanism, while the N–S–S-donor Ru(II) complex (**Ru2**) performed cell death via necroptosis. Particularly, the direct effect of the straight-chain hydrocarbon groups in the structure of **Ru1** on the activity was observed. The weak activity of **Ru1** (particularly Bax and Bcl2) in Vero cells may indicate that this complex is selective for cancer cells. **Ru1** and **Ru 2** exhibited better immunohistochemical activity than **Ir1**. In conclusion, Ru(II)–arene complexes may be an alternative to platinum-based anticancer drugs.

Berndsen et al. reported the angiostatic and antitumor activity of a drug combination containing RAPTA-C and erlotinib. Immunohistochemical staining for the endothelial cell marker CD31 and the proliferation marker Ki67 was evaluated on A2780 and A2780cisR cell lines. The drug combinations inhibited the tumor growth up to 67%.^30^ Montani et al. synthesized a cationic Ru(II) arene complex and reported that it suppressed triple negative breast cancer growth by inhibiting tumor infiltration of regulatory T cells by immunohistochemical analysis.^31^ Liang et al. synthesized polypyridine ruthenium(II) complexes and investigated their anticancer efficacy both *in vitro* and *in vivo*. Immunohistochemical analysis results showed that the complex prevented tumor growth and induced apoptosis, as indicated by Ki67 staining and TUNEL staining, respectively, in comparison to the positive control.^32^ In the literature, there are studies that include histological staining involving Ru(II) arene complexes. However, we have not encountered a study where both apoptosis and necroptosis markers were simultaneously applied and analyzed. Our study is unique in this regard, as it clearly demonstrates how the mechanism of cell death changes when different metal ions bind to the same ligand structure.

#### 2.2.4 Stability of the Complexes

**Figure 9:**
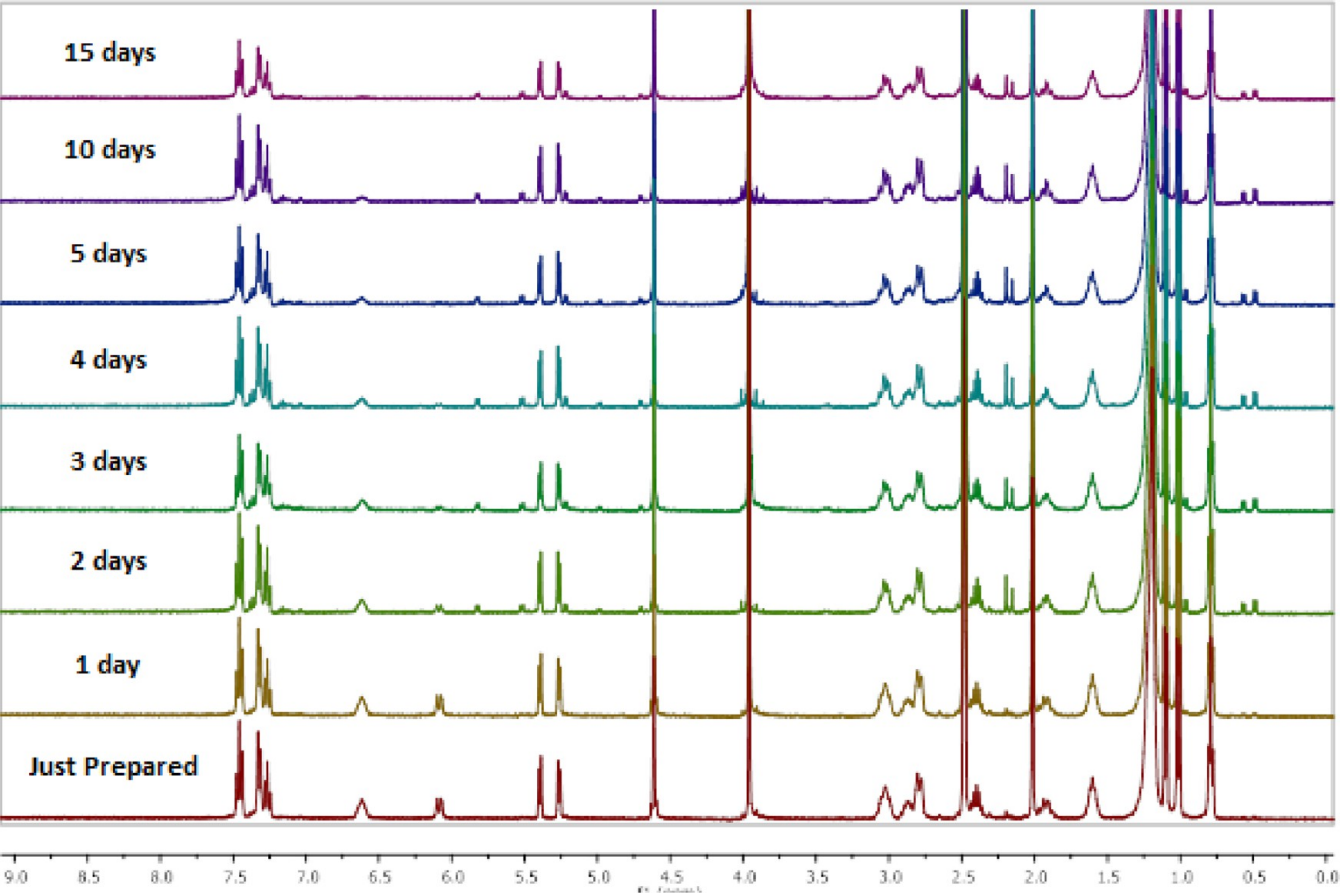
Stability of **Ru1** was monitored via ^1^H NMR spectroscopy in 20% D_2_O/DMSO-*d*_6_ over 15 days.

Overall, a complete understanding of the chemical properties and behavior of metal complexes as well as careful experimental design and execution is crucial for ensuring accurate and reliable *in vitro* studies. Therefore, we must carefully consider the choices of solvent and concentration for *in vitro* studies with metal complexes and to evaluate the stability of the complex under specific conditions. This may also help to compare the stability of the complex in DMSO with that in other solvents or to evaluate the stability using multiple methods, such as NMR spectroscopy or electrochemical analysis, to ensure reliability of results. NMR spectroscopy can be used to determine the coordination geometry of the metal center and to monitor changes in the chemical environment of the ligands. Thus, we adopted time-dependent stability to identify the stability of **Ru1** in the D_2_O/DMSO-*d*_6_ (20:80) solvent system via ^1^H NMR spectroscopy (Figure 9). We observed that the complex remained stable in solution for up to 2 days, which included the incubation period. After the second day, the NH protons, which were seen around 6 and 6.5 ppm, gradually disappeared. This disappearance could be due to deuterium exchange from D_2_O.

**Figure 10:**
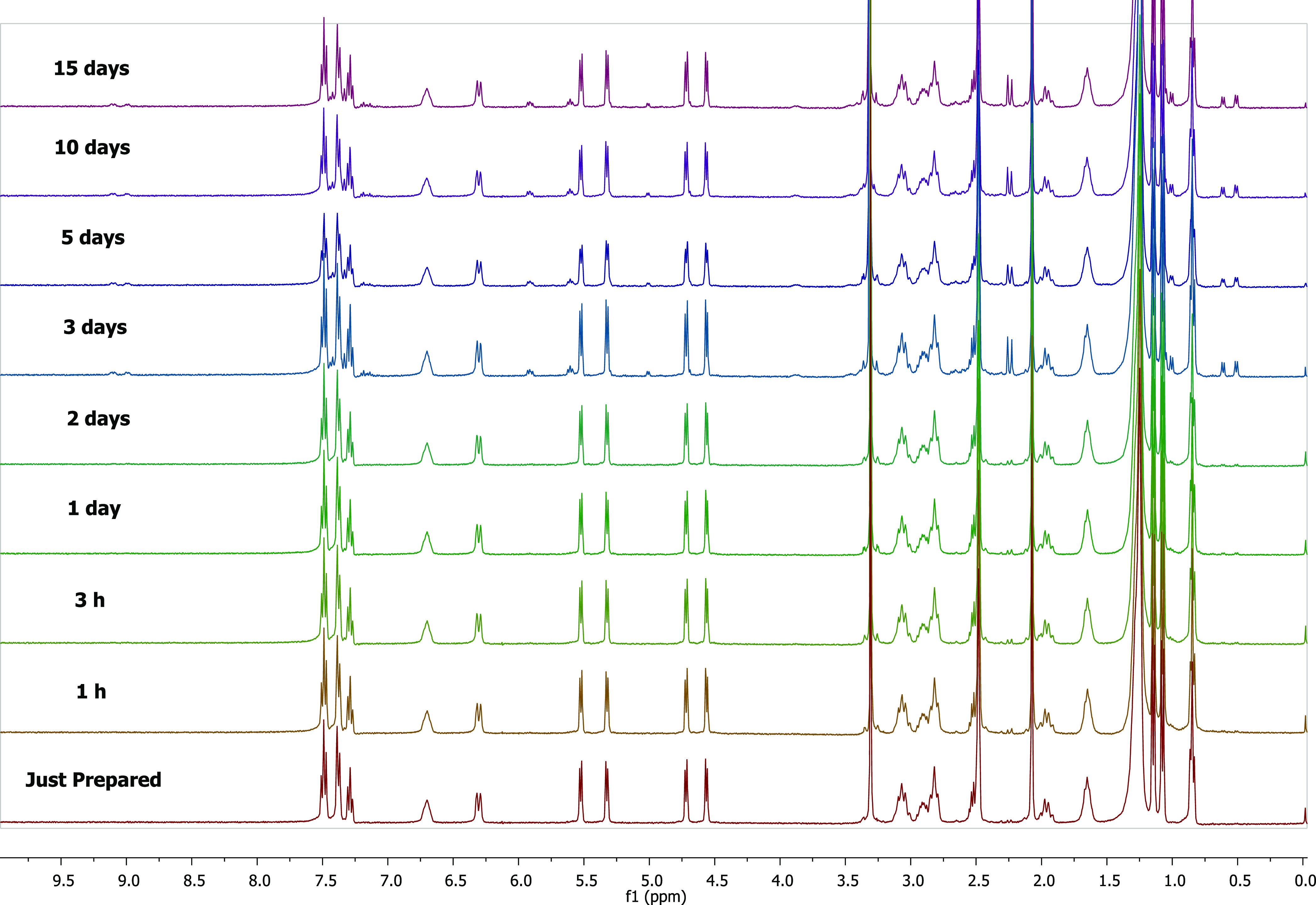
Stability of **Ru1** was monitored via ^1^H NMR spectroscopy in DMSO-*d*_6_ over 15 days.

We also analyzed stability in 100% DMSO-*d*_6_ solvent (Figure 10). The complex retained its structure for up to 15 days. Unlike in aqueous solution, NH protons did not disappear. In the analyses performed after the 15th day, no specific changes were observed. A new type of complex is likely to form in the solution. When we analyze the spectrum taken on day 15, we observe that the proportion of this new species is 3.55%. However, we did not take it into account because this ratio is very low, and these new peaks were not observed during the incubation process, indicating no effect on the biological activity.

#### 2.2.5 Interaction with FS-DNA

**Figure 11:**
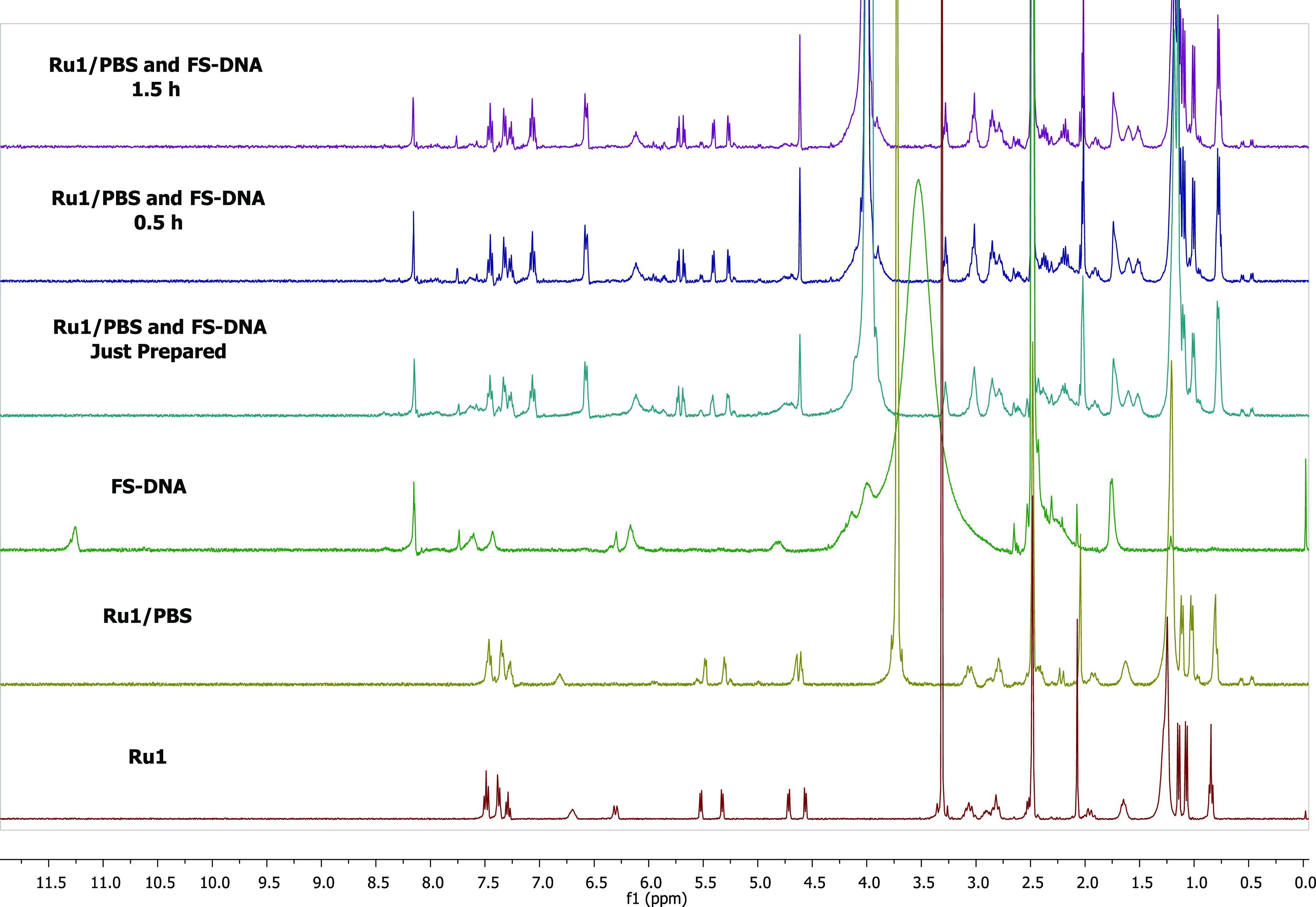
Interaction of **Ru1** with FS-DNA was monitored via ^1^H NMR spectroscopy in DMSO-*d*_6_/D_2_O over 90 min.

DNA is the primary target for the potential antitumor activity of much of the ruthenium(II) arene complexes. For this purpose, interaction studies with DNA are important to elucidate the apoptosis mechanism of the complex.^33^ We investigated the interaction of **Ru1** with FS-DNA by NMR spectroscopy (Figure 11). First, **Ru1** was analyzed in DMSO-*d*_6_ solution. Then, 0.01 M PBS was added to the first solution and two drops of D_2_O added to better dissolve PBS. The pH was adjusted to 7.4. FS-DNA was also analyzed in DMSO-*d*_6_. FS-DNA solution and complex/PBS solution were combined and kept at 39 °C and analyzed over 90 min.

The detailed ^1^H NMR spectrum and structure of FS-DNA are presented in Figure S30. Upon addition of FS-DNA to the complex solution, the peaks at 11.0–11.5 ppm in FS-DNA disappeared immediately. This peak belongs to the carboxylic acid proton of FS-DNA. The disappearance of this peak indicates that the complex binds to FS-DNA via the oxygen atom of the carboxylic acid. Changes in the chemical shifts and splitting of the peaks after FS-DNA addition are clearly seen. A singlet is observed at 4.5 ppm after the combination of the complex and FS-DNA. This is the CH proton bound to carbon 8 of FS-DNA (see Figure S30), which was at 4 ppm before the association and shifted to 4.5 ppm afterward. The possible structure resulting from the interaction is shown in Figure S31.

In recent years, platinum-based agents have been widely used in chemotherapeutic treatment.^34^ However, interest in other metal complexes is on the rise owing to the toxicity of platinum-based agents and the increased drug resistance of diseased cells.^35^ Some Ru compounds are highly selective for cancer cells and, thus, exhibit low toxicity.^36^ A previous study by Sadler et al. revealed the success of RAED-type Ru(II)–arene complexes.^37^ Particularly, the high activity of the RM175 complex demonstrated the importance of the arene group and ethylenediamine ligand.^38^ We studied the activity of RAED complexes substituted with different aromatic and aliphatic groups,^10^ and in this study, we have presented a novel, RAED-type complex (**Ru1**). Ru(II)–arene complexes with N and P donors have been widely investigated in literature, whereas studies on complexes with S donors are somewhat limited.^39^ Henderson et al. reported the cytotoxic activities of N,S-coordinated mono- and bimetallic organo-rhodium(III), -Ir(III) and -Ru(II) complexes on A549 cells.^40^ Thiolato-bridged, dinuclear, arene–Ru complexes are the most promising biological agents to substitute cisplatin in future cancer therapy strategies.^41^ Complexes that contain different aromatic and benzylic groups attached to the sulfur atom are being increasingly investigated. In some cases, an increase in the number of metal centers does not positively affect the cytotoxic activity. With increasing molecular weight, solubility and intracellular transport may decrease, and the interaction of the complex with the target can weaken due to steric hindrance. However, in dimeric complexes, since the bond between the bridging atoms is weak, this bond can be disrupted in the solvent, and the complex can symmetrically split into two, allowing solvent molecules to bind to the coordination gaps. This contributes positively to the solubility of the complex. Therefore, although there are bimetallic complexes (**Ru2** and **Ir1**) in our study, their solubility is good due to their dimer structure.

**Figure 12:**
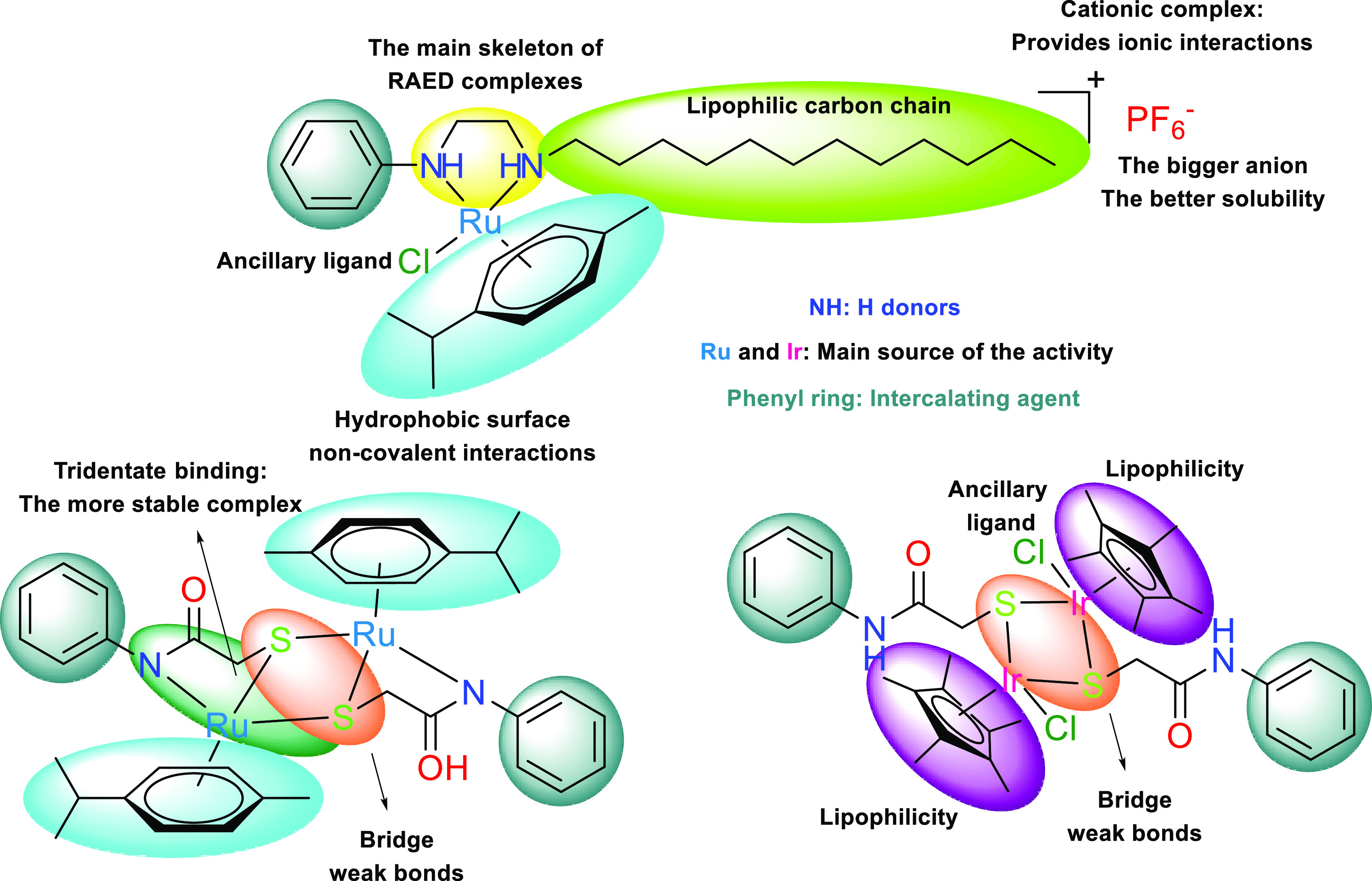
Illustration of structure–activity relationships of the complexes (**Ru1**, **Ru2**, and **Ir1**).

**Figure 13:**
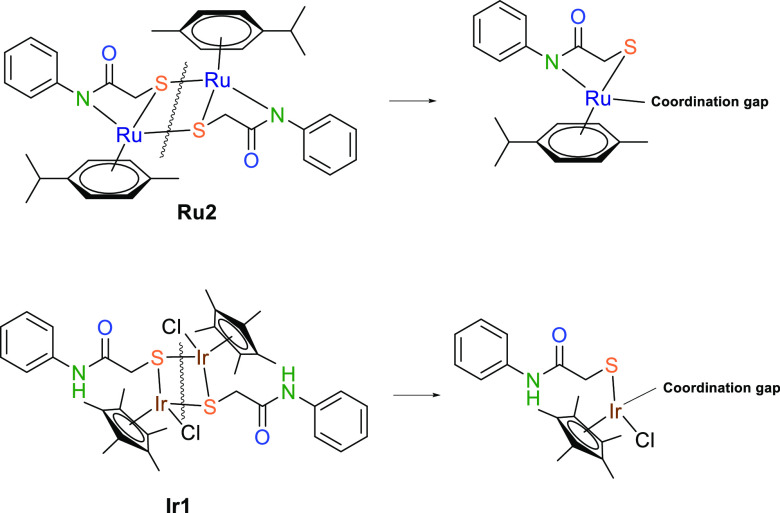
Dissociation of the dimeric complexes in a solution.

The structure–activity relationship (SAR) of Ru(II) arene complexes reflects the correlation between their structural features and biological activities. Understanding this relationship is essential for designing Ru(II) arene complexes with desired properties for medicinal chemistry.^42,43^ SAR data of the complexes (**Ru1**, **Ru2**, and **Ir1**) are presented in Figure 12. **Ru1** represents a classic RAED-type Ru(II) arene complex. The main target of RAED-type complexes is DNA. Being a cationic complex, **Ru1** can perform ionic interactions with DNA or other target biomolecules. The solubility of a complex in various solvents or biological environments is essential for its practical use, especially in biological applications. An important feature of **Ru1** is its straight carbon chain structure, enhancing lipophilicity for efficient cell membrane penetration. The easily detachable chloride ion of **Ru1** enables interactions with proteins or DNA base pairs within the cell. Additionally, two hydrogen bond donors in its structure contribute to these interactions. On the other hand, **Ru2** and **Ir1** are dimeric neutral complexes with the same ligand system. **Ru2** features tridentate N,S,S donor atoms for metal coordination, while **Ir1** contains bidentate S,S donor atoms. This coordination difference results in **Ru2** lacking a readily detachable ancillary ligand. However, the dimeric structure of **Ru2** suggests the possibility of forming a coordination gap upon the bridging bond’s cleavage (Figure 13). **Ir1** benefits from two cyclopentadienyl (Cp*) rings, imparting high lipophilicity. Understanding the structural features and their impact on the activity of Ru(II) arene complexes allows researchers to design complexes with tailored properties for specific applications. It also enables the optimization of complexes for improved therapeutic potential, such as the development of novel anticancer agents.

The stability, controlled geometry, and tunable properties of Ru(II) arene complexes containing chelating ligands contribute to their versatility and effectiveness in a wide range of applications. This study is expected to bring a new perspective to the field with the novel ruthenium and iridium complexes it reports.

## 3 Conclusion

We reported a series of mono- and bimetallic Ru(II)–*p*-cymene complexes (**Ru1 and Ru2**) and bimetallic Ir(III)–pentamethylcyclopentadienyl complex (**Ir1**). **Ru2** and **Ir1** unexpectedly formed bimetallic, dimeric structures. **Ru1** was weak in Bax, Bcl2, and caspase-3 immunoreactivities in HepG2 cells and negative in Vero cells. **Ru2** was observed to be moderate in caspase-3, RIP3, and RIPK1 immunoreactivities in both cell lines, whereas **Ir1** mainly exhibited an increase in RIPK1 immunoreactivity. We observed that **Ru1** activates apoptosis, particularly in HepG2 cells, whereas **Ru2** and **Ir1** have an effect on activating necroptosis by increasing RIPK1 immunoreactivity in both cell lines. **Ru1** was confirmed to remain stable in solution for up to 15 days, and its interaction with FS-DNA was monitored by ^1^H NMR spectroscopy. These unexpected structures and the different coordinating natures of metals are exciting in the context of inorganic coordination chemistry.

## 4 Experimental Section

### 4.1 Chemistry

#### 4.1.1 General Information

Reactions that involved air-sensitive components were performed using a Schlenk-type flask under argon atmosphere and high-vacuum line techniques. The glass equipment was heated under vacuum to remove oxygen and moisture, after which it was filled with argon. Ir(III) chloride hydrate, thioglycolic acid, toluene, potassium carbonate, and 1,2,3,4,5-pentamethylcyclopentadiene were obtained from Merck; Ru(III) chloride hydrate was obtained from abcr; α-terpinene was obtained from Sigma; aniline was obtained from Alfa Aesar; acetonitrile and dimethyl sulfoxide were obtained from Carlo Erba. ^1^H, ^13^C, 2D ^1^H–^1^H gHSQC, and 2D ^1^H–^13^C gCOSY NMR spectra were recorded on a Varian 400 MHz spectrometer. XRD analysis was performed on a D8-QUEST diffractometer equipped with graphite-monochromatic Mo–Kα radiation or a STOE IPDS-II diffractometer using graphite-monochromated Mo–Kα radiation via the ω-scan method. FT-IR spectra were recorded on a PerkinElmer Spectrum 100 series instrument. Melting points (m.p. values) were measured on an Electrothermal IA9100 melting point apparatus. RuCl_2_(*p*-cymene)]_2_ was prepared according to the method reported by Bennett and Smith through the reaction of Ru(III) chloride with α-terpinene.^44^ [IrCl_2_Cp*]_2_ was synthesized according to the published procedures via a reaction between Ir(III) chloride and pentamethylcyclopentadiene.^45^

#### 4.1.2 General Procedure for the Synthesis of 2-Mercapto-*N*-phenylacetamide (**A**)

Aniline (0.91 mL, 10.00 mmol) and thioglycolic acid (0.69 mL, 10.0 mmol) were dissolved in 5 mL of dry toluene. The resulting mixture was stirred at 120 °C overnight under argon atmosphere, after which it was cooled to room temperature. The resulting white precipitates were filtered and washed with hexane. Yield: 1.40 g (84%), white powder, m.p.: 168 °C. ^1^H NMR (400 MHz, DMSO-*d*_6_): δ 10.15 (s, 1 H, N-*H*), 7.58 (d, *J* = 8.4 Hz, 2 H, P-*H*), 7.31 (t, *J* = 7.6 Hz, 2 H, Ph-*H*), 7.06 (t, *J* = 7.2 Hz, 1 H, P-*H*), and 3.72 (s, 2 H, C*H*_2_). ^12^C NMR (100 MHz, DMSO-*d*_6_): δ 167.14, 139.19, 129.22, 124.01, 119.72, and 43.64.

#### 4.1.3 General Procedure for the Synthesis of **Ru1**

**Ru1** was prepared according the published procedure. L (1.00 g, 3.28 mmol) and [RuCl_2_(*p*-cymene)]_2_ (1.00 g, 1.64 mmol) were dissolved in 20 mL of dry MeCN. The mixture was refluxed overnight. NH_4_PF_6_ (0.53 g, 3.28 mmol) was dissolved with MeCN and added to the mixture. The mixture was filtered. The filtrate was evaporated to dryness. The crude solid product was purified with column chromatography. Yield: 0.90 g (76%), yellow powder, m.p.: 93–95 °C. Anal. Calcd for C_30_H_50_ClF_6_N_2_PRu (720,23): C, 50.03; H, 7.00; N, 3.89. Found: C, 49.86; H, 6.98; N, 3.73. ^1^H NMR (400 MHz, DMSO-*d*_6_): δ 7.51 (t, *J* = 7.6 Hz, 2 H, Ar-*H*), 7.39 (d, *J* = 7.6 Hz, 2 H, Ar-*H*), 7.31 (t, *J* = 7.6 Hz, 1 H, Ar-*H*), 6.70 (br, 1 H, N*H*), 6.32 (br, 1 H, N*H*), 5.53 (d, *J* = 6.0 Hz, 1 H, *p*-cymene-Ar-*H*), 5.33 (d, *J* = 6.0 Hz, 1 H, *p*-cymene-Ar-*H*), 4.72 (d, *J* = 6.0 Hz, 1 H, *p*-cymene-Ar-*H*), 4.57 (d, *J* = 6.0 Hz, 1 H, *p*-cymene-Ar-*H*), 3.09 (m, 2 H, NCH_2_C*H*_2_N), 2.93 (m, 1 H, C*H*_2_), 2.85 (m, 2 H, NC*H*_2_CH_2_N), 2.51 (m, 1 H, *p*-cymene-C*H*), 2.07 (s, 3 H, *p*-cymene-C*H*_3_), 2.00 (m, 1 H, C*H*_2_), 1.68 (m, 2 H, C*H*_2_), 1.25 (s, 20 H, C*H*_2_), 1.15 (d, *J* = 6.8 Hz, 3 H, *p*-cymene-C*H*_3_), 1.08 (d, *J* = 7.2 Hz, 3 H, *p*-cymene-C*H*_3_), and 0.86 (t, *J* = 6.8 Hz, 3 H, C*H*_3_). ^13^C NMR (100 MHz, DMSO-*d*_6_): δ 150.34, 129.57, 126.28, 106.66, 94.70, 86.14, 82.93, 81.26, 79.91, 56.37, 52.54, 48.54, 31.77, 30.41, 29.54, 29.49, 29.46, 29.20, 28.24, 26.96, 22.74, 22.58, 21.51, 17.17, and 14.45.

#### 4.1.4 General Procedure for the Synthesis of **Ru2**

**A** (0.10 g, 0.60 mmol) and K_2_CO_3_ (0.16 g, 1.20 mmol) were dissolved in 5 mL of dry acetonitrile. The mixture was stirred for half an hour at room temperature. RuCl_2_(*p*-cymene)]_2_ (0.19 g, 0.30 mmol) was added to this reaction mixture. The final mixture was stirred overnight at room temperature. Upon filtering the mixture, the filtrate was evaporated to dryness. The crude solid product was purified via column chromatography (dichloromethane/methanol). The precipitate was recrystallized in DCM/Et_2_O. Yield: 0.20 g (32%), orange crystal, m.p.: 252 °C. Anal. Calcd for C_36_H_42_N_2_O_2_Ru_2_S_2_ (801,00): C, 53.98; H, 5.29; N, 3.50; S, 8.00. Found: C, 53.63; H, 5.25; N, 3.27; S, 8.12. ^1^H NMR (400 MHz, CD_3_OD): δ 7.41 (m, 4 H, Ph-*H*), 7.18 (m, 1 H, Ph-*H*), 5.52 (d, *J* = 6.0 Hz, 1 H, *p*-cymene-Ar-*H*), 5.37 (d, *J* = 6.0 Hz, 1 H, *p*-cymene-Ar-*H*), 5.05 (d, *J* = 6.0 Hz, 1 H, *p*-cymene-Ar-*H*), 4.94 (d, *J* = 6.0 Hz, 1 H, *p*-cymene-Ar-*H*), 3.56 (q, *J* = 15.6–48.0 Hz, 2 H, C*H*_2_), 2.00 (s, 3 H, *p*-cymene–C*H*_3_), 1.93 (m, 1 H, *p*-cymene-C*H*), 1.07 (d, *J* = 6.8 Hz, 3 H, *p*-cymene-C*H*_3_), and 0.85 (d, *J* = 6.8 Hz, 3 H, *p*-cymene-C*H*_3_). ^13^C NMR (100 MHz, CD_3_OD): δ 178.77, 153.08, 128.37, 126.59, 124.33, 107.11, 100.60, 88.34, 84.58, 83.08, 81.96, 43.24, 30.01, 22.08, 20.61, and 16.81.

#### 4.1.5 General Procedure for the Synthesis of **Ir1**

**A** (0.10 g, 0.60 mmol) and [IrCl_2_Cp*]_2_ (0.24 g, 0.30 mmol) were dissolved in 10 mL of acetonitrile. The mixture was stirred overnight at room temperature. Upon evaporating the mixture to dryness, the crude solid product was purified via column chromatography (dichloromethane/methanol). The resulting precipitate was recrystallized in DCM/Et_2_O. Yield: 0.20 g (63%), yellow crystal, m.p.: 284 °C. Anal. Calcd for C_36_H_46_Cl_2_Ir_2_N_2_O_2_S_2_ (1058,23): C, 40.86; H, 4.38; N, 2.65; S, 6.06. Found: C, 40.51; H, 4.28; N, 2.58; S, 6.17. ^1^H NMR (400 MHz, CDCl_3_): δ 8.77 (s, 1 H, N-*H*), 7.63 (d, *J* = 8.0 Hz, 2 H, Ph-*H*), 7.35 (t, *J* = 8.0 Hz, 2 H, Ph-*H*), 7.16 (t, *J* = 7.2 Hz, 1 H, Ph-*H*), 3.98 (s, 2 H, C*H*_2_), and 1.61 (s, 15 H, Cp*C*H*_3_). ^13^C NMR (100 MHz, CDCl_3_): δ 166.16, 137.43, 129.01, 124.85, 120.07, 44.41, and 8.82.

#### 4.1.6 XRD Analysis

X-ray single-crystal diffraction data for **Ru2** was collected at room temperature on a STOE IPDS-II diffractometer using graphite-monochromated Mo–Kα radiation via the ω-scan method. Data collection and cell refinement were performed using X-AREA, while data reduction was performed using X-RED32.^46^ The crystal structure was solved with the ShelXT^47^ solution program using dual methods and using Olex2^48^ as the graphical interface. The model was refined using ShelXL^49^ with full-matrix least-squares minimization on F2. All non-hydrogen atoms were anisotropically refined. Hydrogen atom positions were geometrically calculated and refined using the riding model, fixing the aromatic C–H distances at 0.93 Å, methylene C–H distances at 0.97 Å, methine C–H distances at 0.98 Å, and methyl C–H distances at 0.96 Å. Uiso(H) values were set to 1.2 Ueq (1.5 Ueq for the methyl group) with respect to the parent atom. Figures of molecular structure were created using Olex2.^48^ CCDC: 2259511 contains the supplementary crystallographic data for **Ru2**.

A suitable crystal of **Ir1** was selected for data collection that was performed on a Bruker D8-QUEST diffractometer equipped with graphite-monochromatic Mo–Kα radiation at 296 K. The H atoms were located on different maps and then treated as riding atoms with C–H distances in the range of 0.93–0.97 Å and N–H distances of 0.86 Å. Some high residual electron densities (maxima or minima) and metal center separations were between 0.81 and 1.07 Å. Therefore, these high residual electron densities could not be defined based on the refinement of the structure. We performed the following procedures for our analysis: solving via direct methods; SHELXS-2013;^50^ refinement via full-matrix least-squares methods; SHELXL-2013;^51^ molecular graphics: MERCURY^52^ and solution: WinGX.^53^

### 4.2 Pharmacological/Biological Assays

#### 4.2.1 Cell Culture

HepG2 (ATCC, HB-8065TM) cells were cultured in a RPMI 1640 (Capricorn Scientific, RPMI-HA) culture medium that contained 10% FBS (Gibco, 10270-106), 1% penicillin–streptomycin (Capricorn Scientific, PS-B), and 1% l-glutamine (Capricorn Scientific, GLN-B). Vero (normal kidney) (ATCC, CCL-81TM) cells were cultured in a DMEMF12 (Sigma, D6421) culture medium containing 10% FBS, 1% penicillin–streptomycin, and 1% l-glutamine. Both cell lines were incubated in a humidified atmosphere at 37 °C in 5% CO_2_. When the cells were 80% confluent, they were routinely subcultured using a 0.25% trypsin–EDTA solution (Biochrom, L2143).

#### 4.2.2 MTT (3-(4,5-Dimethylthiazol-2-yl)-2,5-diphenyl-2*H*-tetrazolium bromide) Assay for Cell Morphology and Immunohistochemical Evaluation Analyses

The MTT assay, which is based on the reduction of 3-(4,5-dimethylthiazol-2-yl)-2,5-diphenyltetrazolium bromide to a purple formazan product, was performed to estimate cell viability and growth. To determine the appropriate dose and time, we performed the MTT assay in HepG2 and Vero cell lines. Cell suspensions of both cell lines were first prepared at densities of 1 × 10^4^/mL cells per well of 96-well culture dishes and plated in triplicate. Vero and HepG2 cells were then incubated with the **Ru1**, **Ru2**, and **Ir1** (11.10 μM) complexes and as a control with DMSO (Glentham Life Sciences, GK2245) and cisplatin (positive control: 25 μg/mL/83 μM) for 24 and 48 h. After the treatment using the complexes, a 10 μL MTT (Glentham Life Sciences, GC4568) solution was added into each well, followed by incubation for 4 h at 37 °C in 5% CO_2_. The medium was then discarded, and 50 μL of DMSO was added to each well to dissolve the formazan crystals. The absorbance was immediately measured at 540 nm using an UV–visible spectrophotometer multiplate reader.

#### 4.2.3 Immunocytochemistry Analysis

After determining the appropriate dose, Vero and HepG2 cells were seeded for immunocytochemistry analysis, and the **Ru1, Ru2**, and **Ir1** complexes were applied to the cells. The cells were then fixed with 4% paraformaldehyde (Merck, 30525) and washed thrice with phosphate-buffered saline (PBS, Bioshop, PBS404.100). Later, the cells were treated with Triton X-100 (AppliChem, A4975) on ice for permeabilization and washed with PBS. Afterward, endogenous peroxidase activity was quenched by incubation with 3% H_2_O_2_ (Merck, 107209) for 5 min at room temperature. The cells were then washed with PBS and incubated overnight at 4 °C with the following primary antibodies: anti-Bax (Santa Cruz, sc-526), anti-Bcl2 (Santa Cruz, sc-7382), anticaspase 3 (Novus Biologycals, NB-600-1235), anti-RIP3 (Santa Cruz, sc-374639), and anti-RIPK1 (Bioss Antibodies, bs-5805R-TR). The cells were then incubated with secondary antibodies according to manufacturer’s protocols (Thermo Scientific, TP-125-HL), followed by incubation with diaminobenzidine (Thermo Scientific, TA-125-HD) for 5 min to visualize immunolabeling. Following this, the cells were washed using PBS and counterstained using Mayer’s hematoxylin (Sigma-Aldrich, 1.07961.0100) for 1 min and mounted using a mounting medium (Merck Millipore-107961, Germany). All specimens were then evaluated under a light microscope (Olympus BX40, Tokyo, Japan).

## Supporting Information Available

^1^H and ^13^C NMR spectra of **A**; ^1^H, ^13^C, 2D ^1^H–^1^H gHSQC and ^1^H–^13^C gCOSY NMR spectra of **Ru1–2** and **Ir1**; FTIR spectra of **Ru1–2** and **Ir1**; crystal data and structure refinement parameters for **Ru2** and **Ir1**; immunohistochemical analysis results; FS-DNA structure analysis (PDF)
