# Supplementary material for: Cell Death Mechanism of Organometallic Ruthenium(II) and Iridium(III) Arene Complexes on HepG2 and Vero Cells
Source: ACS Omega. 2023 Sep 27;8(40):37549–63. doi: 10.1021/acsomega.3c05898 (PMC10569012; doi:10.1021/acsomega.3c05898)
Supplement: Supplementary file 1 — ao3c05898_si_001.pdf [file ao3c05898_si_001.pdf]

Supporting Information

Cell Death Mechanism of Organometallic Ruthenium(II) and Iridium(III) Arene Complexes on HepG2 and Vero Cells

Serdar Batıkan Kavukcu[1], Hilal Kabadayı Ensarioğlu[2], Hande Karabıyık[3], Hafize Seda Vatansever[2,4], Hayati Türkmen[1*]

[1] Ege University, Faculty of Science, Department of Chemistry, Izmir, 35100, Turkey.

[2] Manisa Celal Bayar University, Faculty of Medicine, Department of Histology and Embryology, Manisa, 45030, Turkey.

[3] Dokuz Eylül University, Faculty of Science, Department of Physics, Izmir, 35390, Turkey.

[4] Near East University, DESAM Institute, Mersin 10, 99138, Turkey.

Contents

^{1}H and ^{13}C NMR spectra of **A**

^{1}H, ^{13}C, 2D ^{1}H-^{1}H gHSQC and ^{1}H-^{13}C gCOSY NMR spectra of **Ru1-2** and **Ir1**

FTIR spectra of **Ru1-2** and **Ir1**

Crystal data and structure refinement parameters for **Ru2** and **Ir1**

Immunohistochemical analysis results

FS-DNA structure analysis

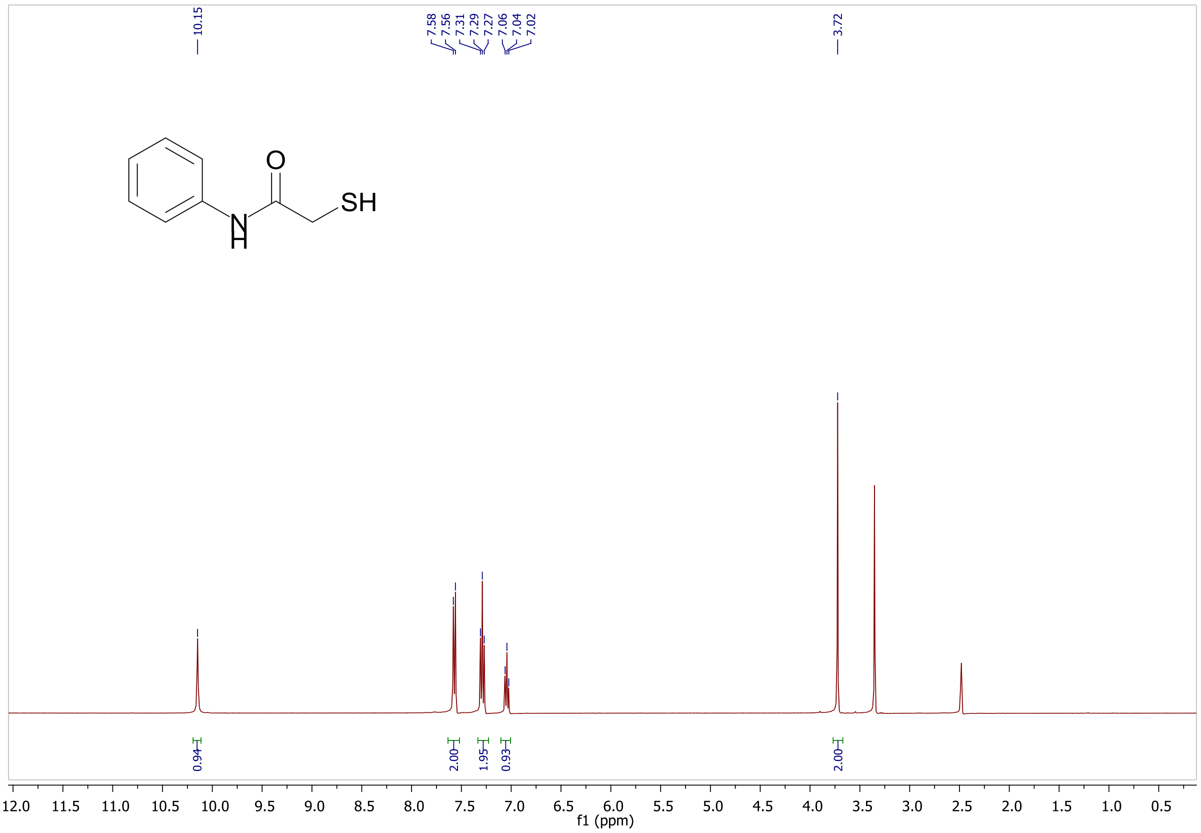

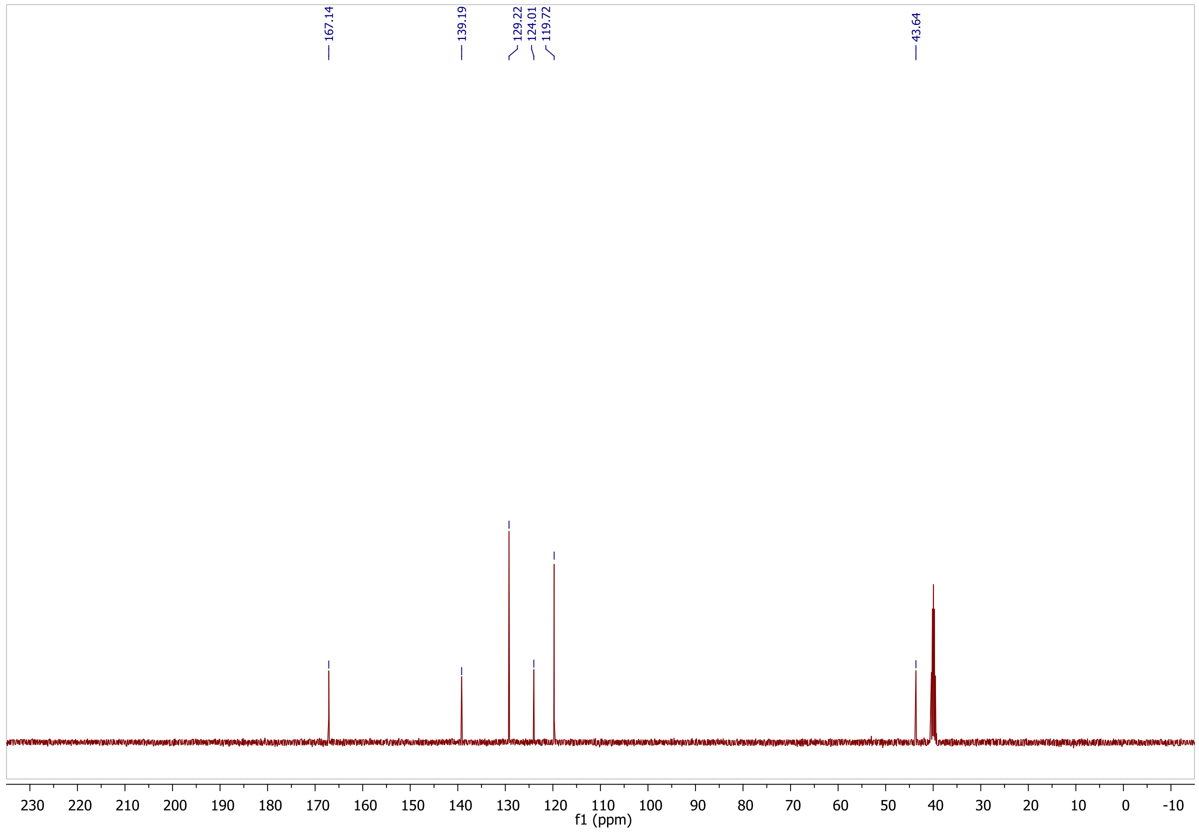

Figure S1. ^{1}H and ^{13}C NMR spectra of **A** (DMSO-d_6).

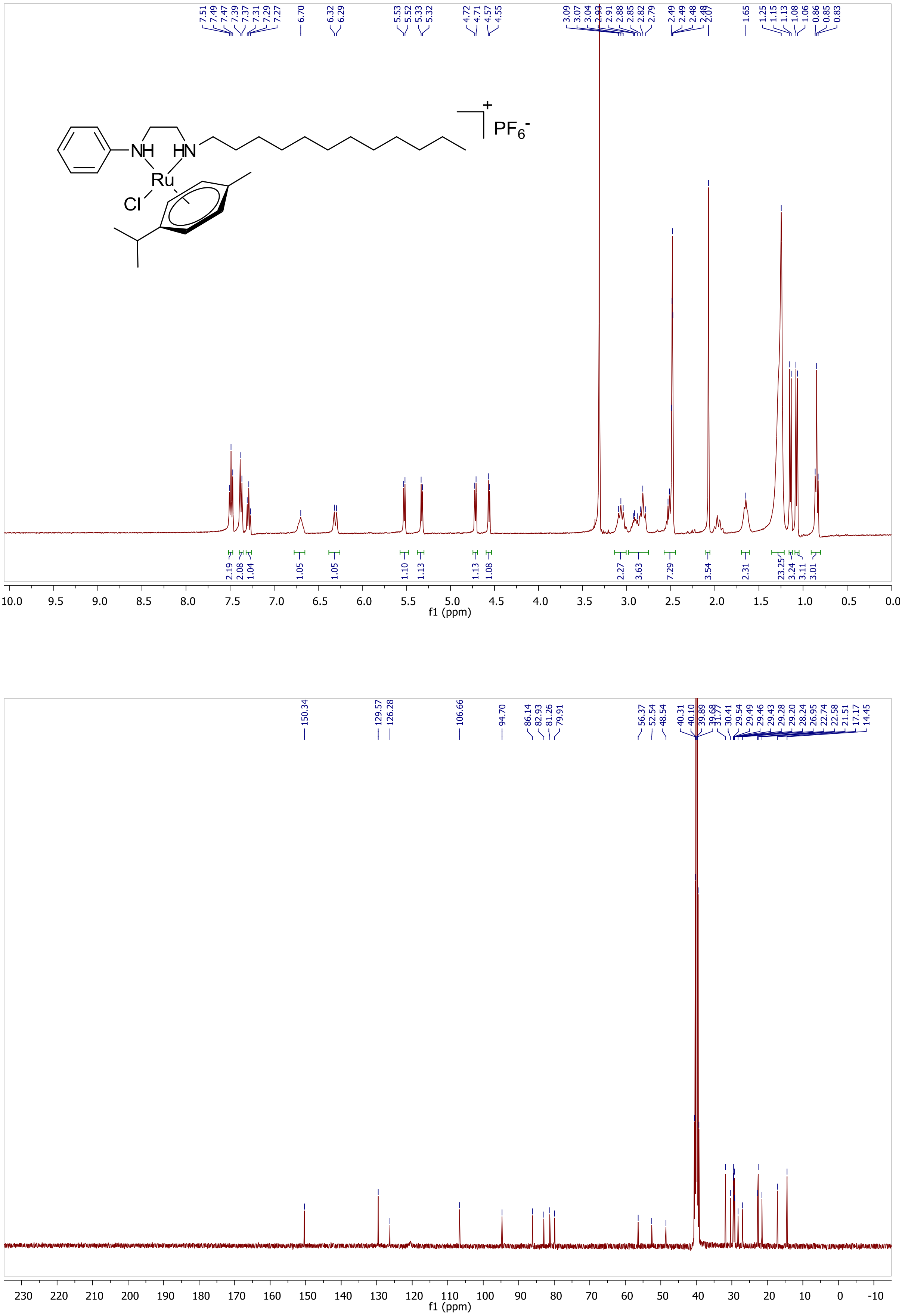

Figure S2. ^{1}H and ^{13}C NMR spectra of **Ru1** (DMSO-d_6).

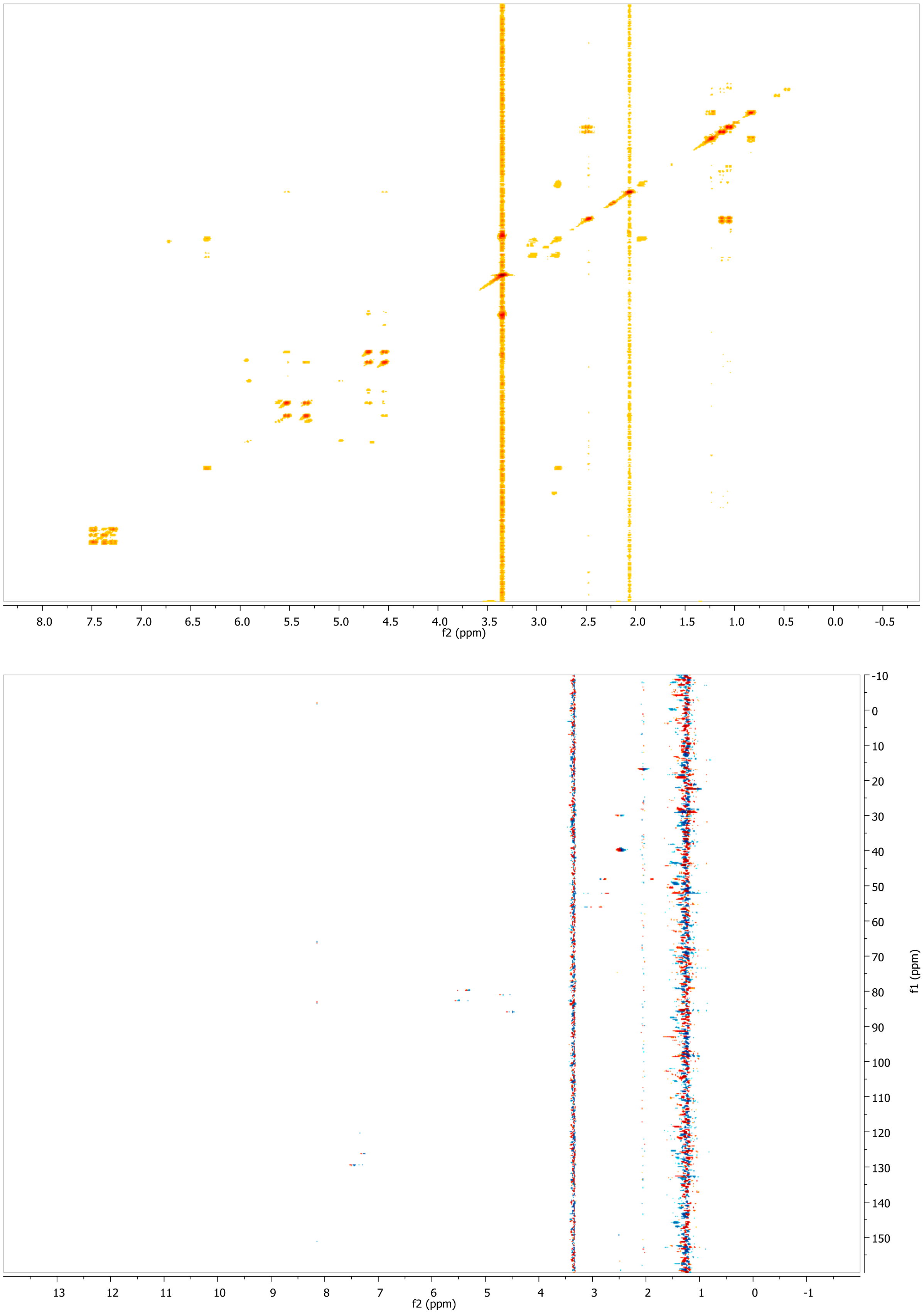

Figure S3. 2D ^{1}H-^{1}H gHSQC and ^{1}H-^{13}C gCOSY NMR spectra of **Ru1** (DMSO-d_6).

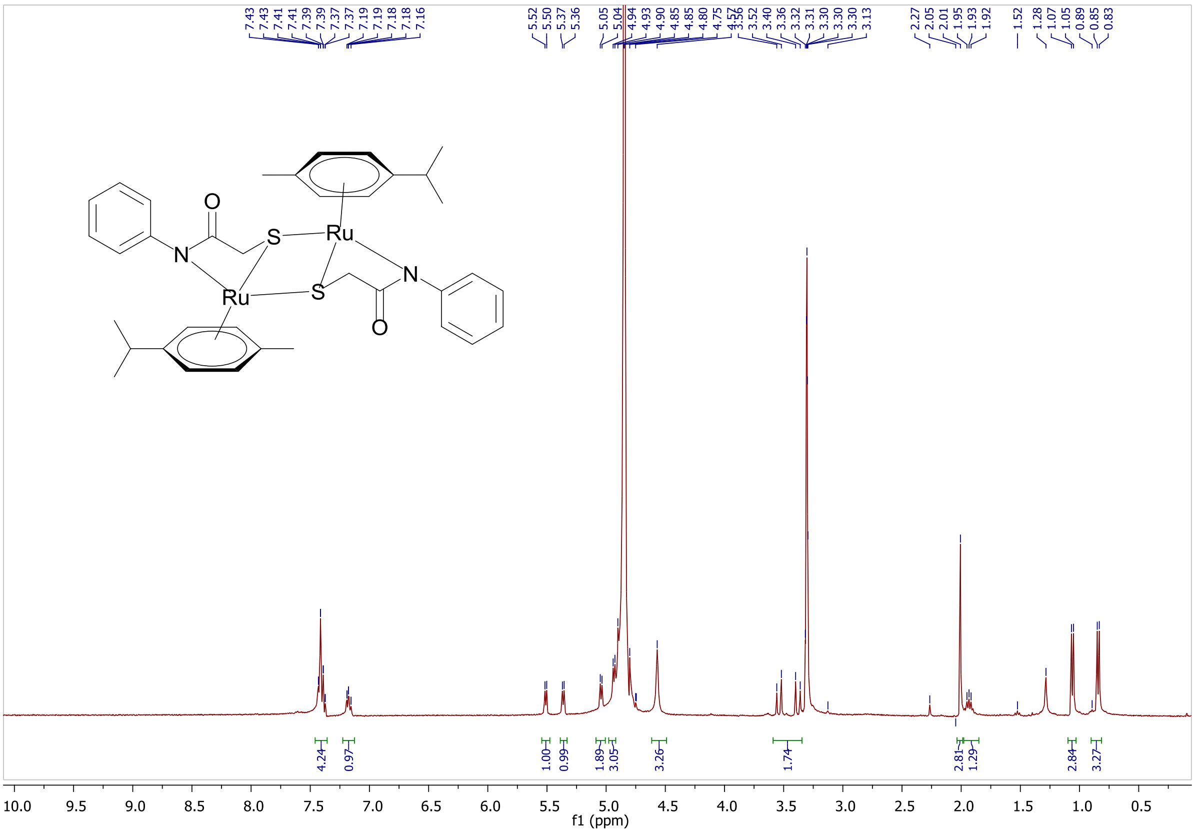

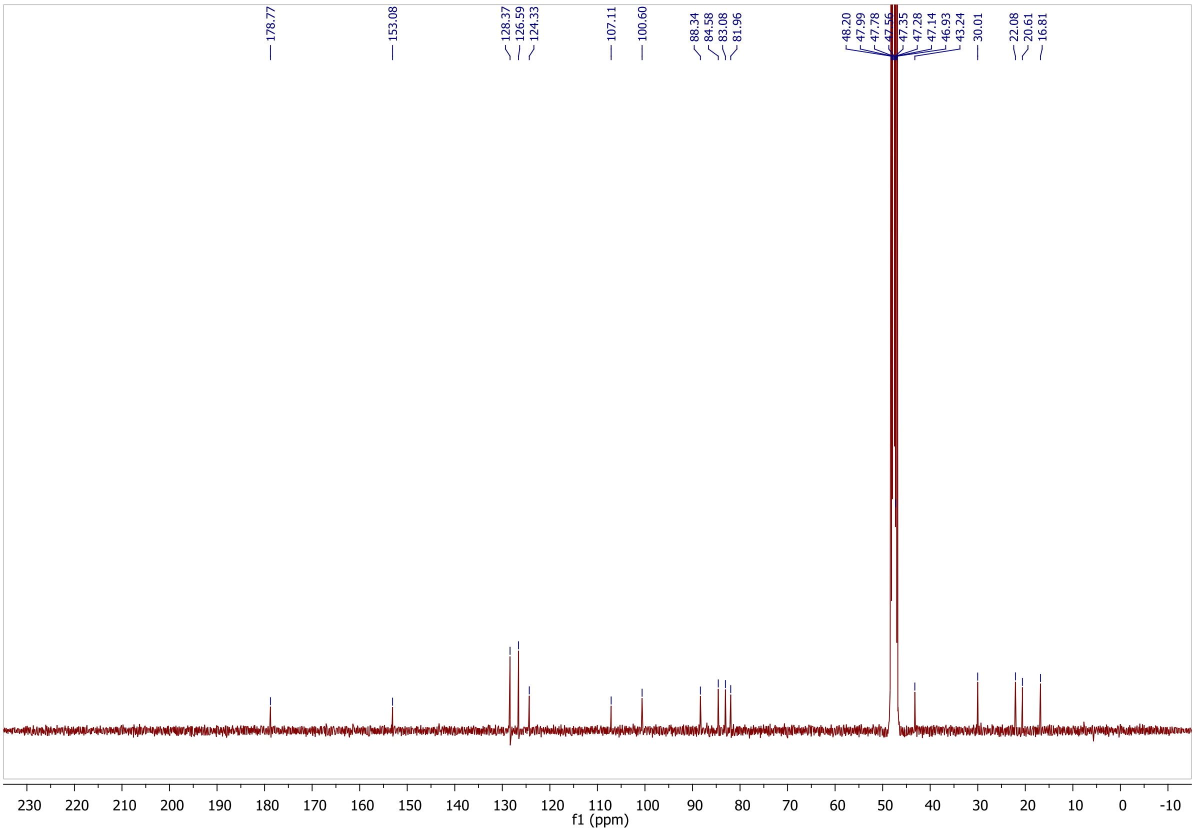

Figure S4. ^{1}H and ^{13}C NMR spectra of **Ru2** (CD_3OD).

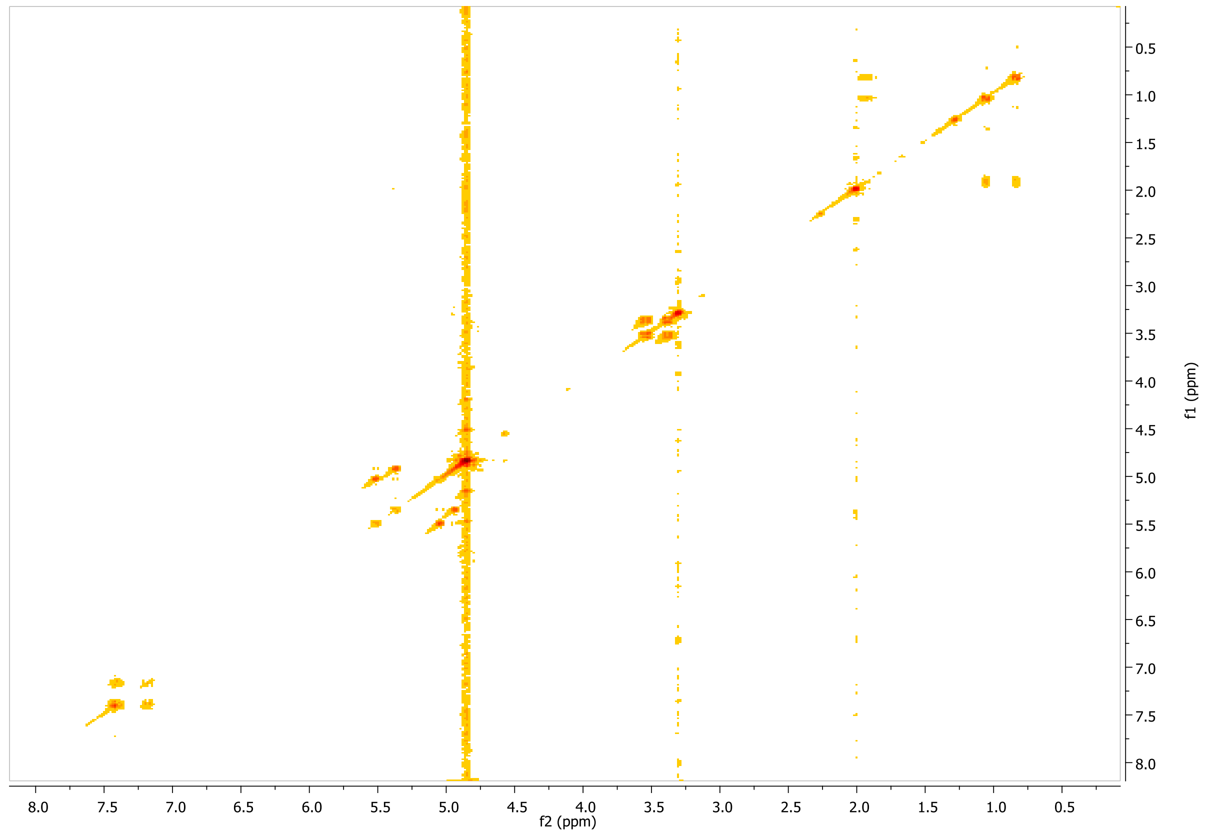

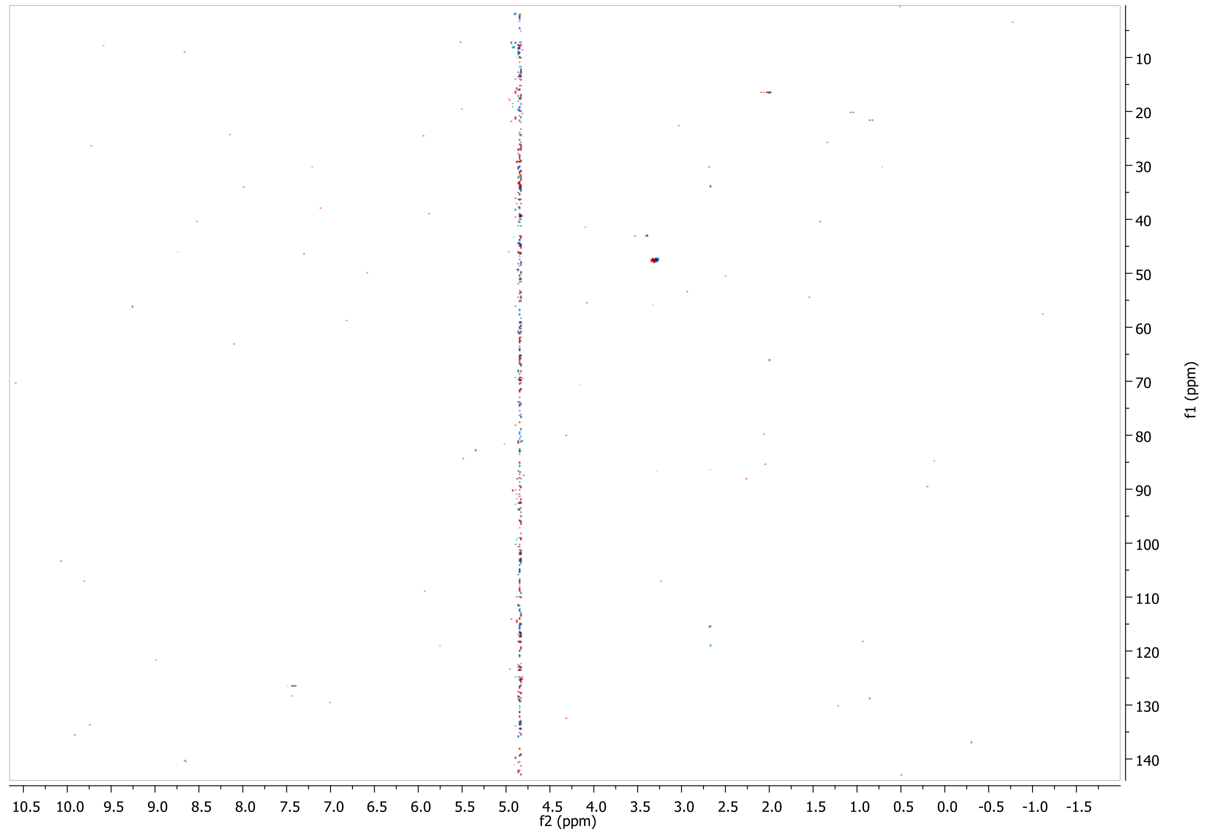

Figure S5. 2D ^{1}H-^{1}H gHSQC and ^{1}H-^{13}C gCOSY NMR spectra of **Ru2** (CD_3OD).

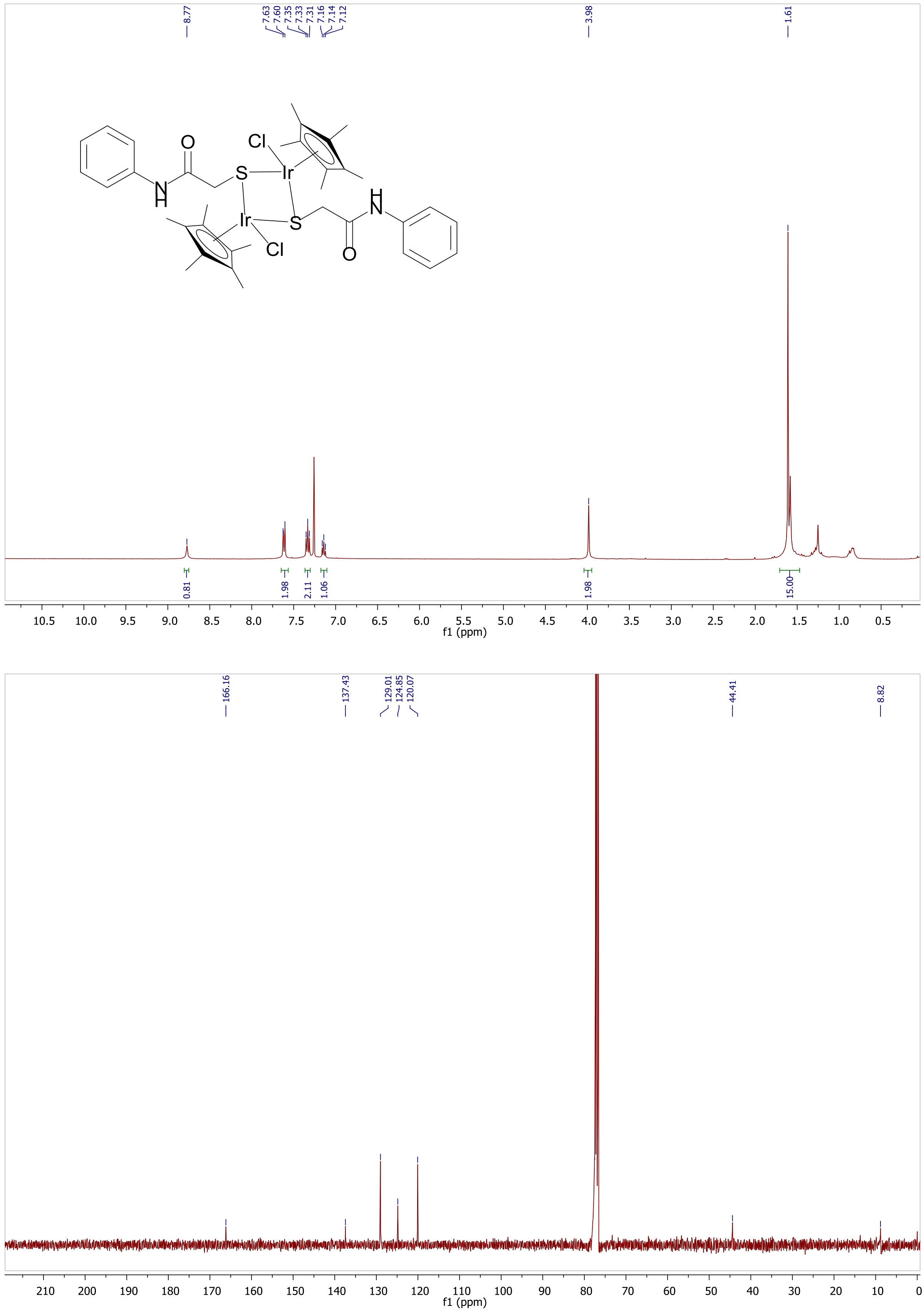

Figure S6. ^{1}H and ^{13}C NMR spectra of **Ir1** ($CDCl_3$).

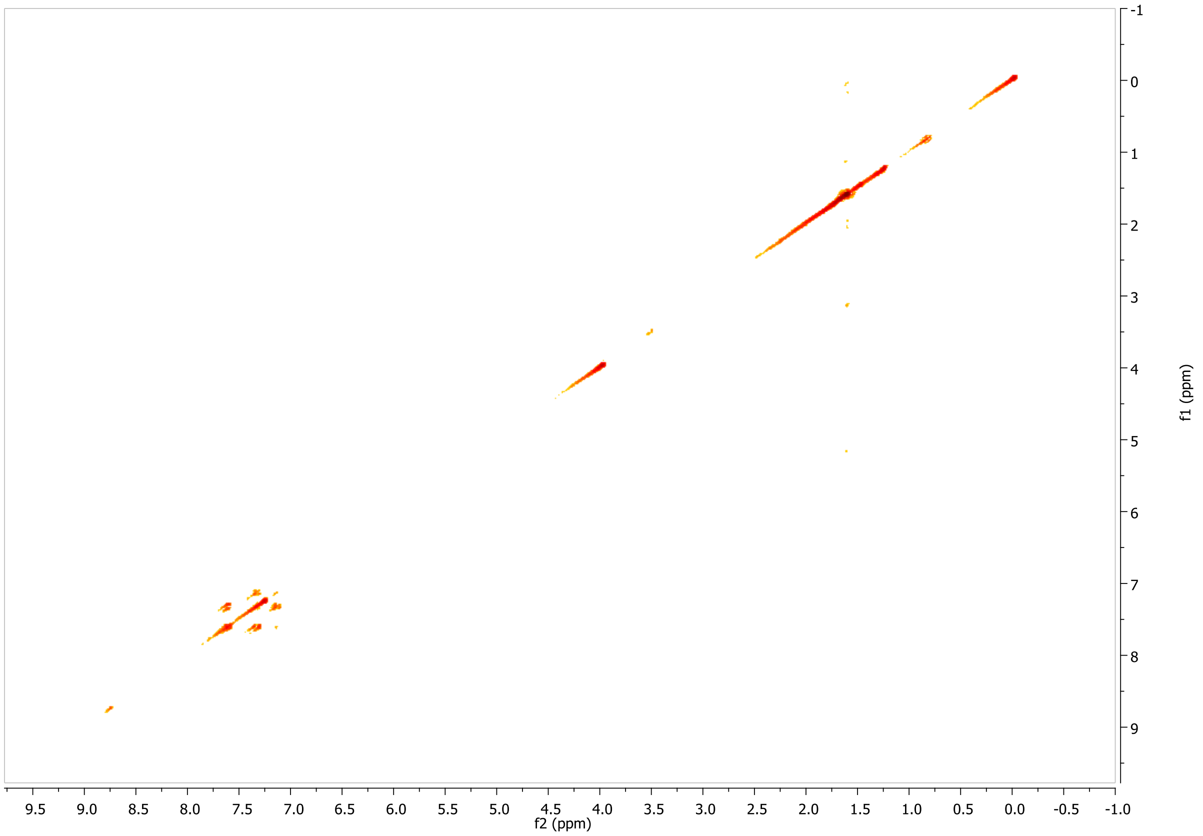

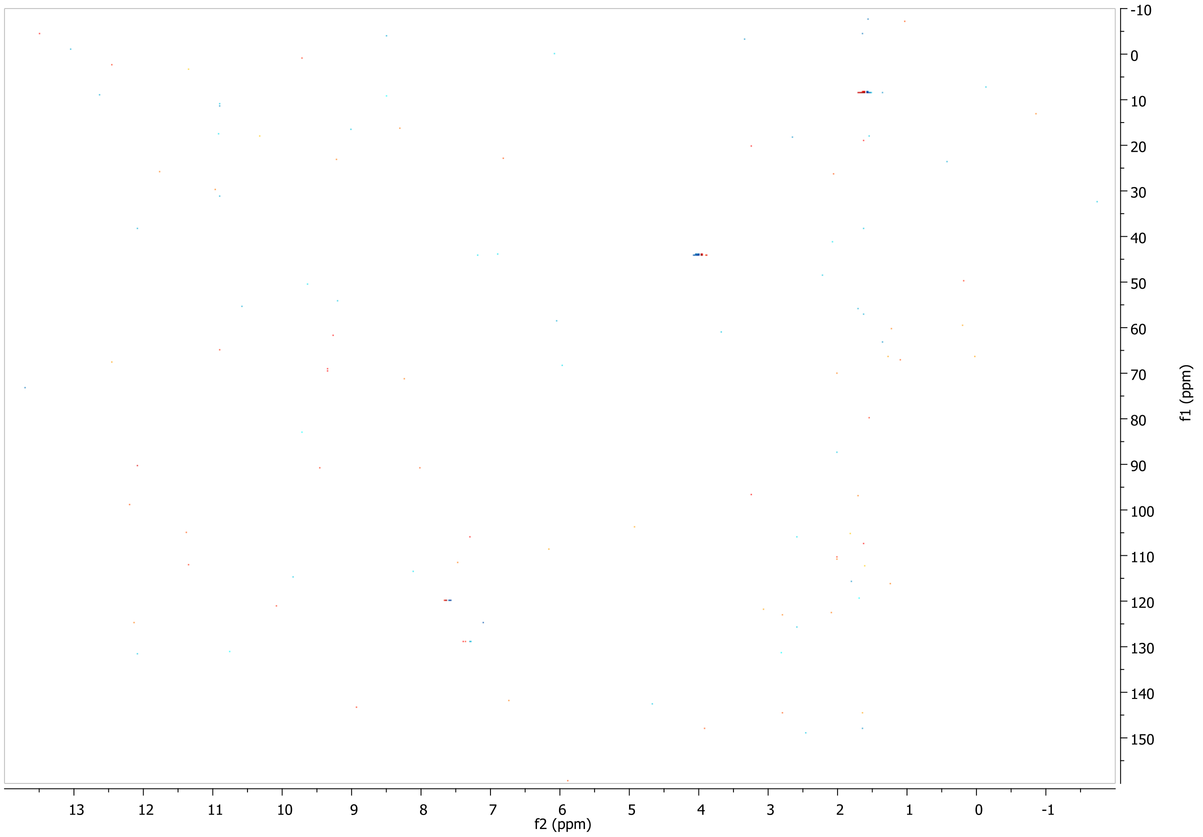

Figure S7. 2D ^{1}H-^{1}H gHSQC and ^{1}H-^{13}C gCOSY NMR spectra of **Ir1** ($CDCl_3$).

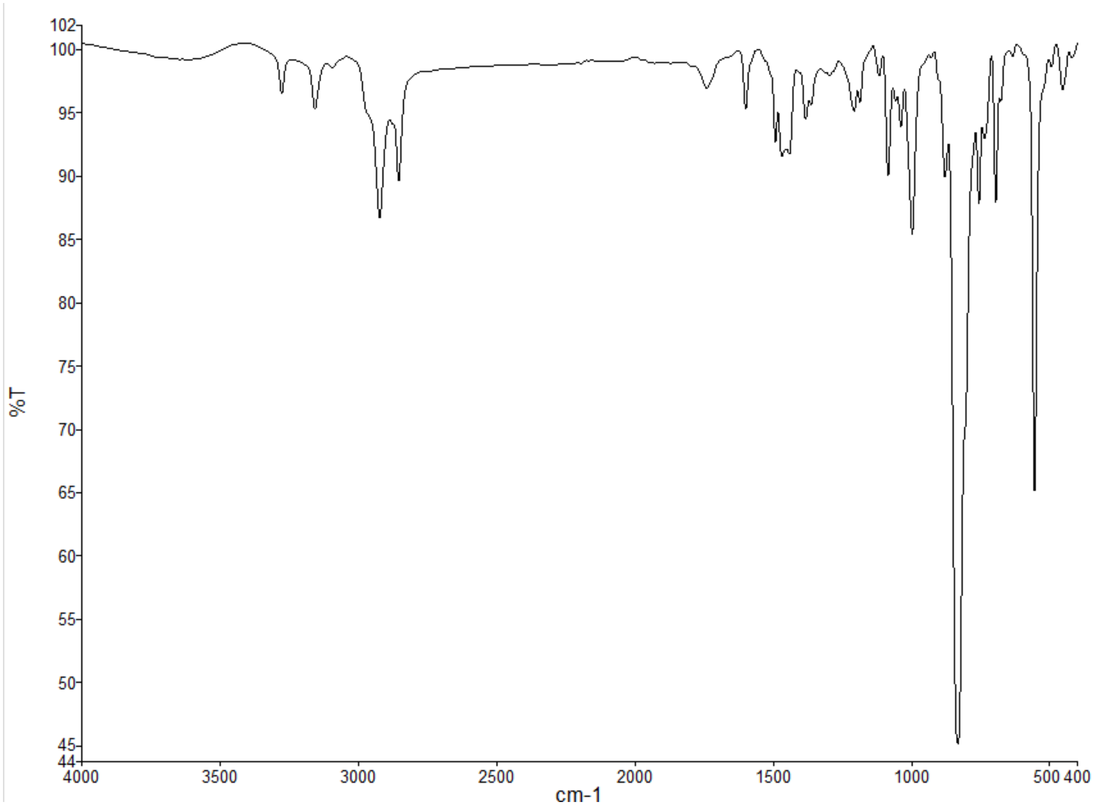

Figure S8. FTIR spectrum of **Ru1**.

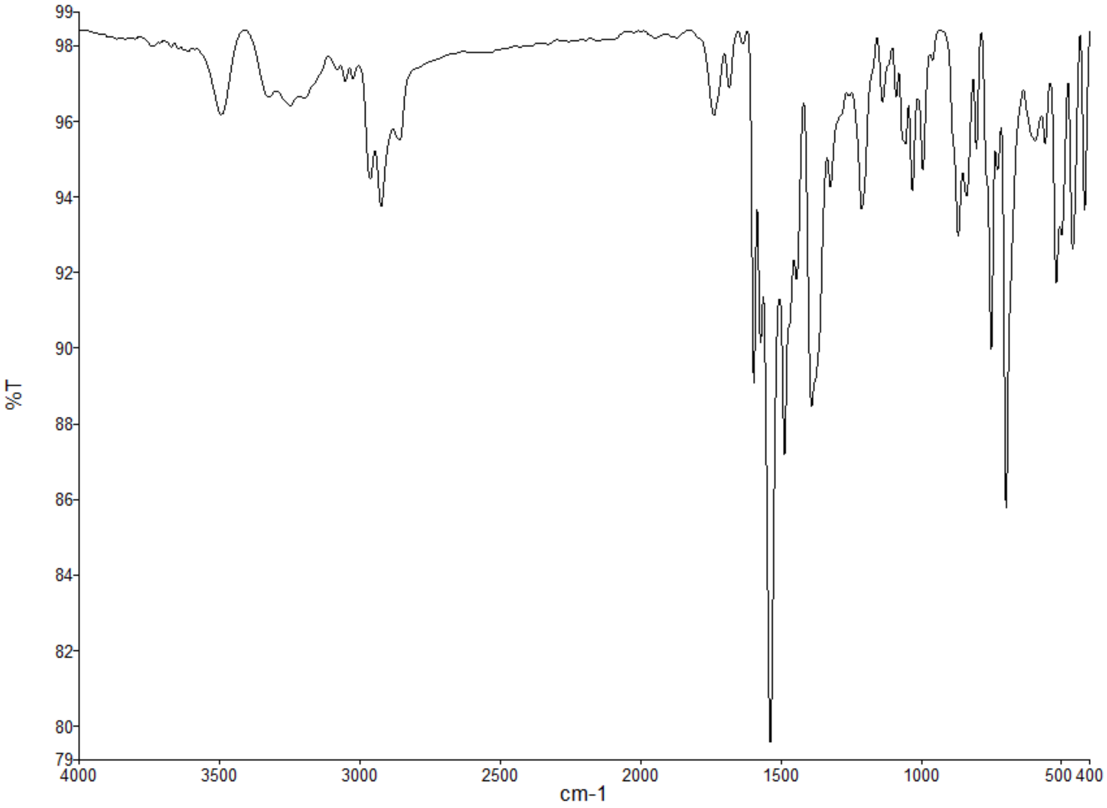

Figure S10. FTIR spectrum of **Ru2**.

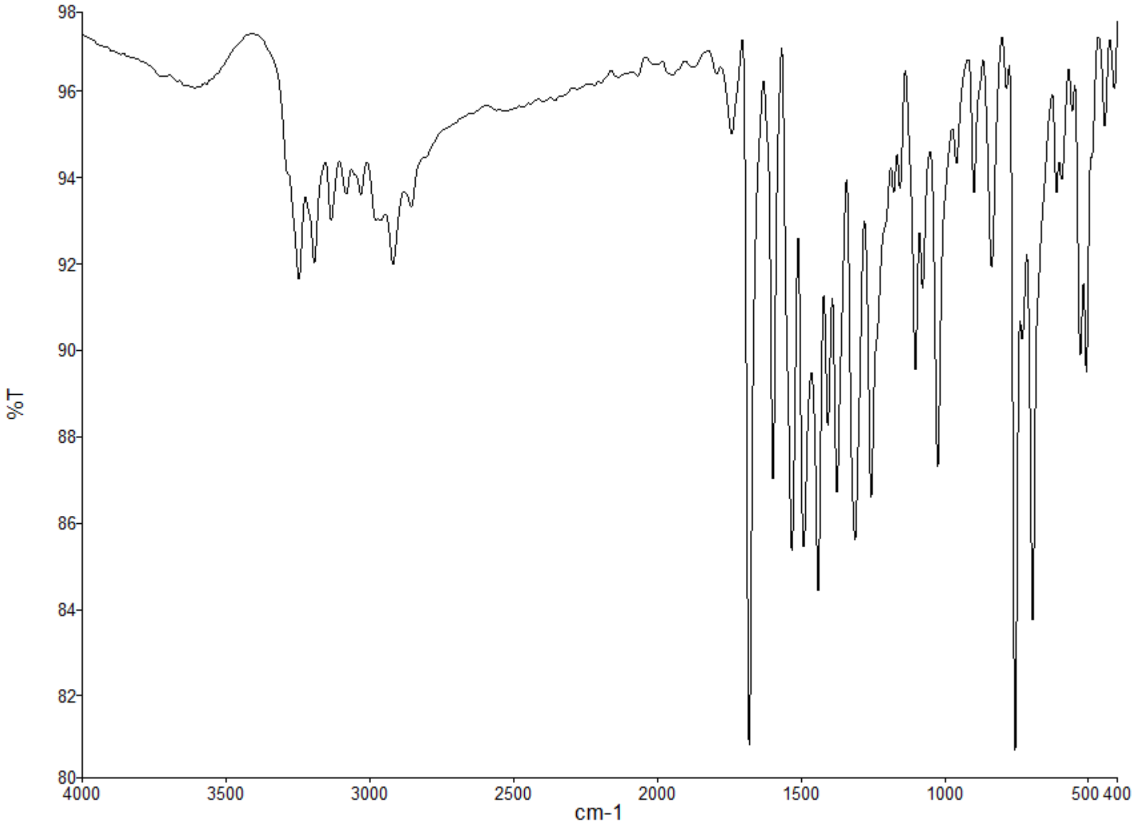

Figure S9. FTIR spectrum of **Ir1**.

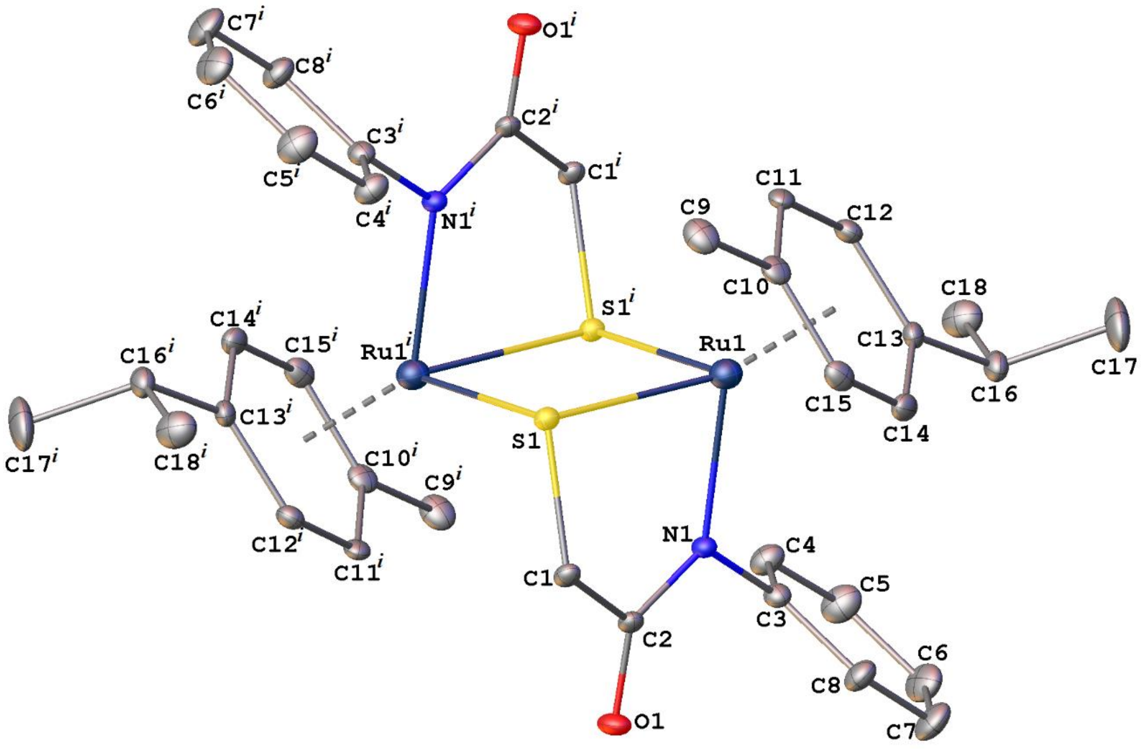

Figure S11. Molecular structure of complex **Ru2** with thermal ellipsoids plotted at the 30% probability level. Symmetry related atoms are labeled with superscript *i*. Symmetry code: (i) 1−*x*, 1−*y*, 1−*z*. Hydrogen atoms are omitted for clarity.

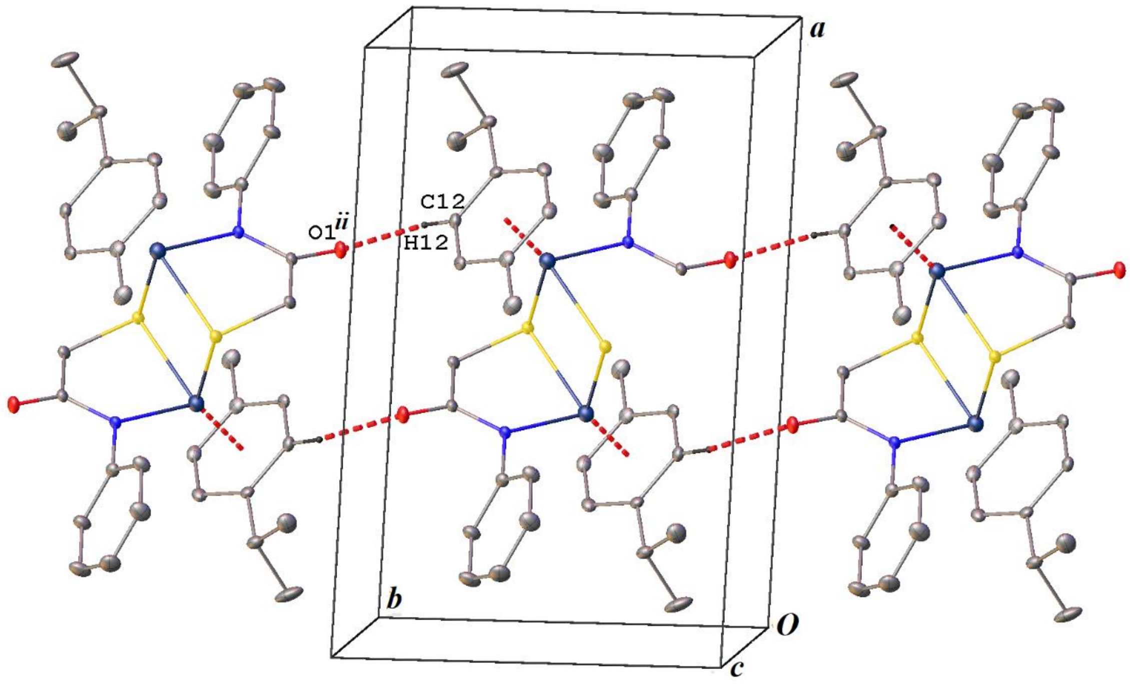

Figure S12. Intermolecular C—H———O hydrogen bonds along the *b* axis of the unit cell. Symmetry code: (ii) *x*, 1+y, *z*. Hydrogen atoms not involved in the interactions have been omitted for clarity.

Table S1. Crystal data and structure refinement parameters for complex **Ru2**.

Empirical formula	$C_{18}H_{21}NORuS$
Formula weight	400.49
Crystal system	Monoclinic
Space group	$P2_1/c$
a (Å)	14.7227(11)
b (Å)	8.6276(4)
c (Å)	15.0760(12)
β(°)	117.063(5)
V (Å^3)	1705.3(2)

Z, Z'	4, 0.5
D_c (g cm^{-3})	1.560
θ range (º)	2.36-27.61
Measured refls.	18754
Independent refls.	3731
R_{int}	0.103
S	1.19
R1/wR2	0.056/0.0937
$\Delta\rho_{max}/\Delta\rho_{min}$ (eÅ^{-3})	0.607/-0.929
CCDC	2259511

Table S2 Selected bond distances and angles for complex **Ru2** (Å, º).

Ru1—S1	2.372 (1)	Ru1—S1^{i}	2.389(1)
Ru1—N1	2.130(4)	S1—C1	1.820(5)
C2—C1	1.522(6)	N1—C2	1.344(6)
O1—C2	1.241(6)	N1—C3	1.442(6)
N1—Ru1—S1	80.78(11)	N1—Ru1—S1^{i}	83.29(11)
C1—S1—Ru1	96.72(16)	C1—S1—Ru1^{i}	108.61(17)
S1—Ru1—S1^{i}	79.06(4)	C2—N1—Ru1	122.1(3)

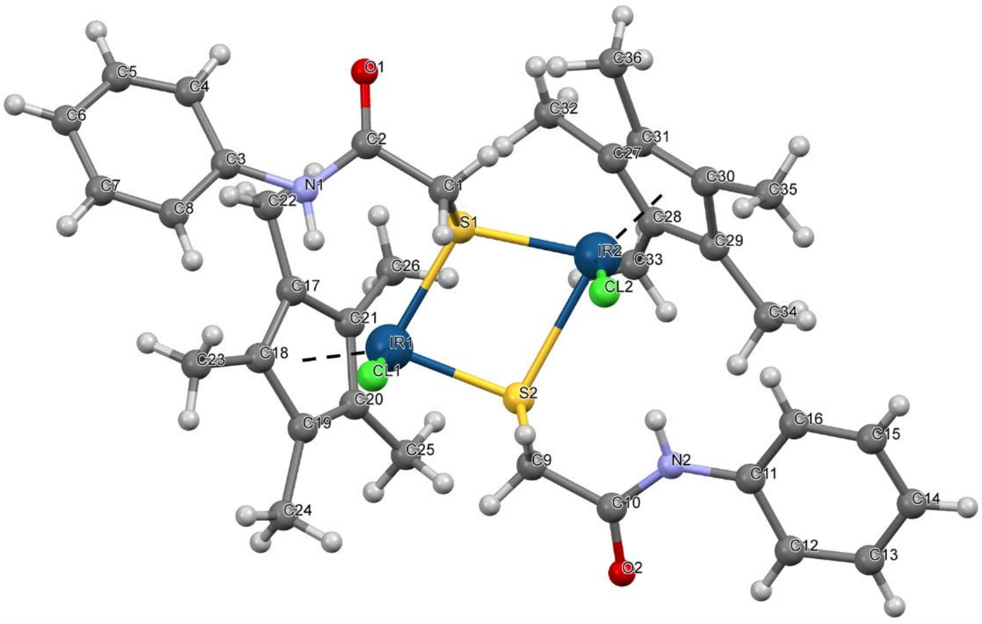

Figure S13. The molecular structure of complex **Ir1** showing the atom numbering scheme.

Table S3. Crystal data and structure refinement parameters for **Ir1**.

Empirical formula	$C_{36}H_{46}Cl_2Ir_2N_2O_2S_2$
Formula weight	1058.17
Crystal system	Orthorhombic
Space group	*Pbca*
a (Å)	16.0948 (9)
b (Å)	15.7661 (10)
c (Å)	30.4609 (19)
V (Å^3)	7729.5 (8)
Z	8

D_c (g cm^{-3})	1.819
θ range (º)	2.7-24.1
Measured refls.	160207
Independent refls.	7165
R_{int}	0.087
S	1.11
R1/wR2	0.105/0.240
$\Delta\rho_{max}/\Delta\rho_{min}$ (eÅ^{-3})	7.95/-3.26
CCDC	2259150

Table S4. Selected bond distances and angles for **Ir1** (Å, º)

Ir1-Cl1	2.410 (5)	Ir2-Cl2	2.407 (5)
Ir1-S1	2.379 (6)	Ir1-S2	2.330 (8)
Ir2-S1	2.363 (7)	Ir2-S2	2.761 (16)
S2-Ir1-S1	81.7 (4)	S2-Ir1-Cl1	95.2 (3)
S1-Ir1-Cl1	92.48 (19)	S1-Ir2-S2	73.5 (2)
S1-Ir2-Cl2	92.6 (2)	S2-Ir2-Cl2	87.8 (2)

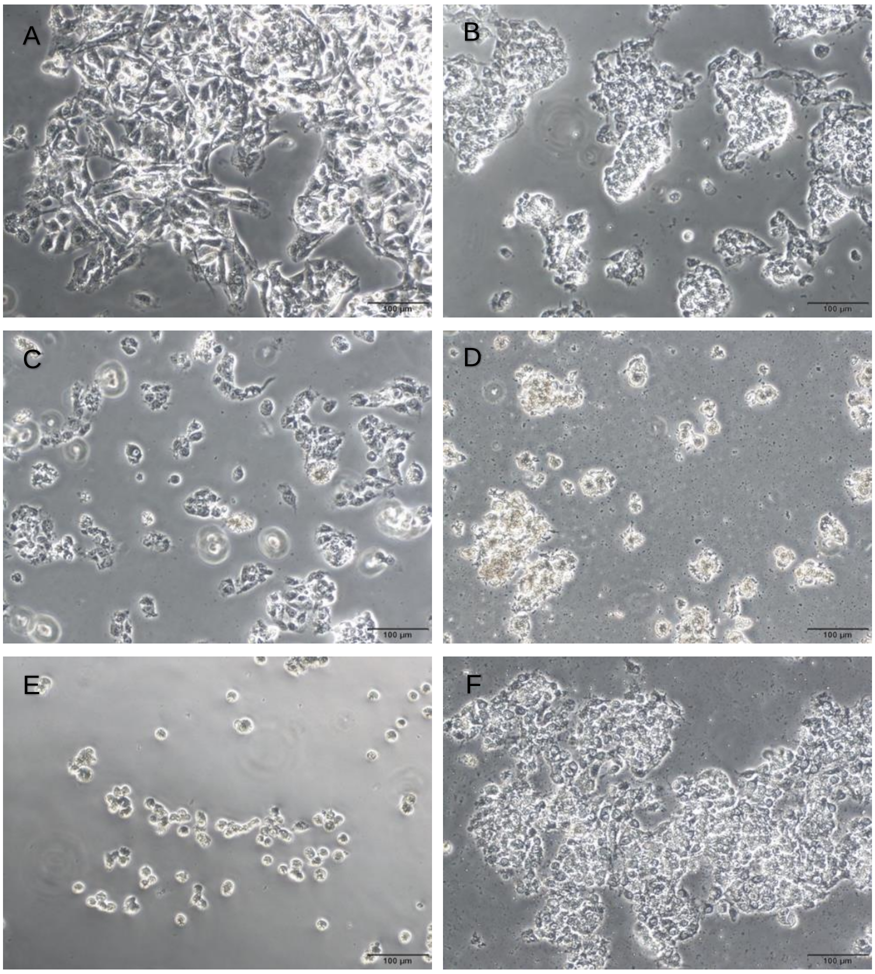

Figure S14. HepG2 cell culture in control (A), DMSO (B), cisplatin (C), **Ru1** applied (D), **Ru2** applied (E) and **Ir1** applied (F) groups after 24 h (left) 48 h (right) of incubation. Scale bars: 100μm

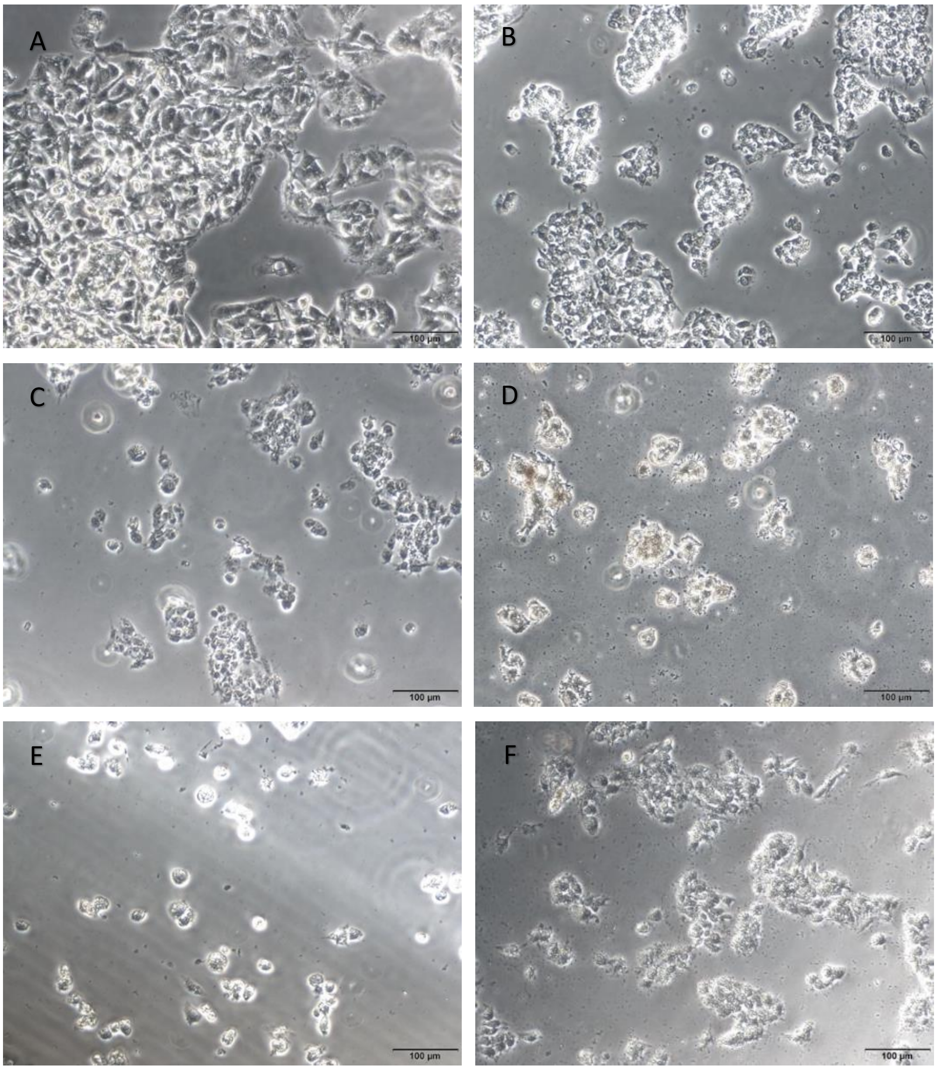

Figure S15. HepG2 cell culture in control (A), DMSO (B), cisplatin (C), **Ru1** applied (D), **Ru2** applied (E) and **Ir1** applied (F) groups after 48 h of incubation. Scale bars: 100μm

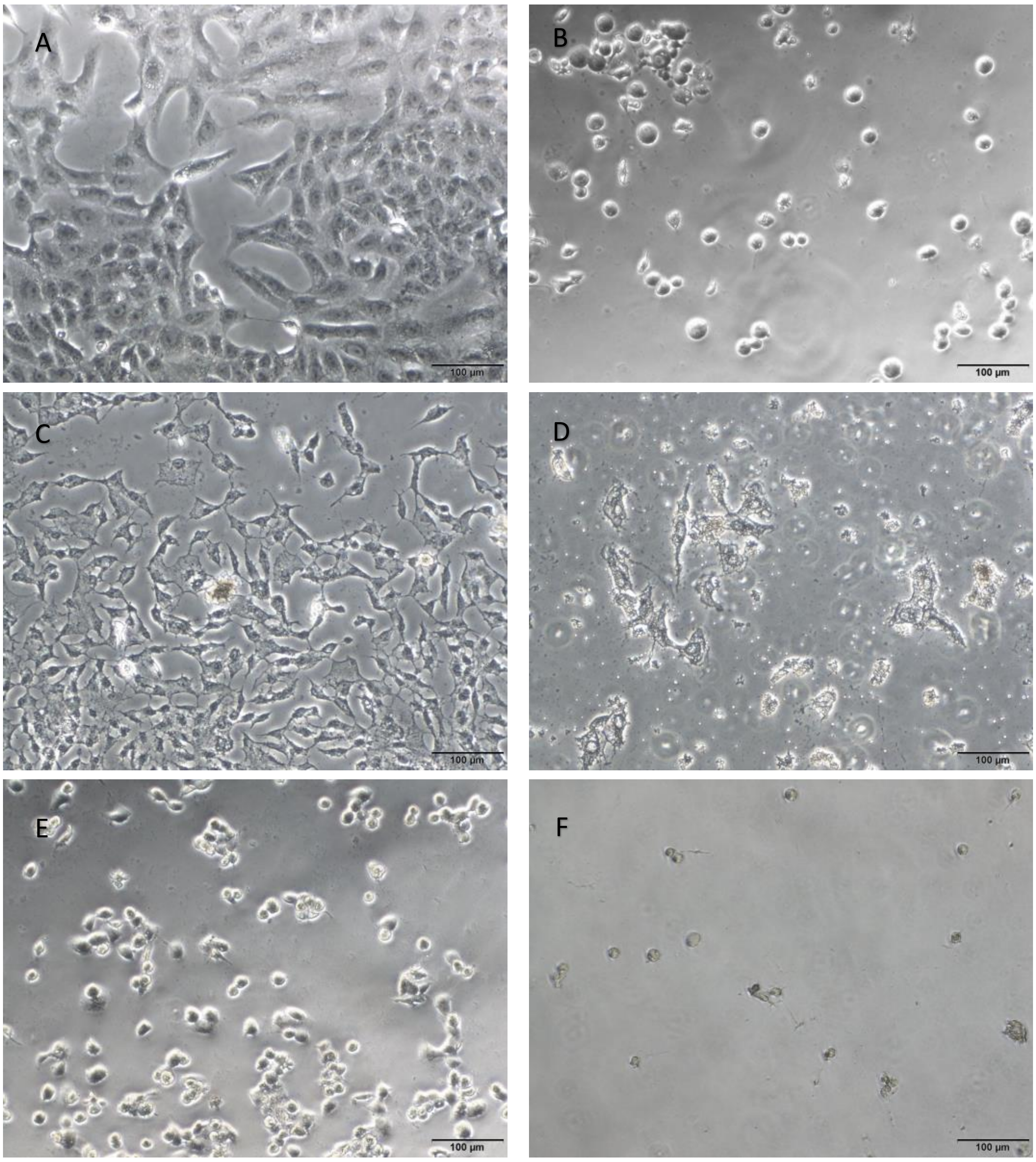

Figure S16. Vero cell culture in control (A), DMSO (B), cisplatin (C), **Ru1** applied (D), **Ru2** applied (E) and **Ir1** applied (F) groups after 24 h of incubation. Scale bars: 100μm

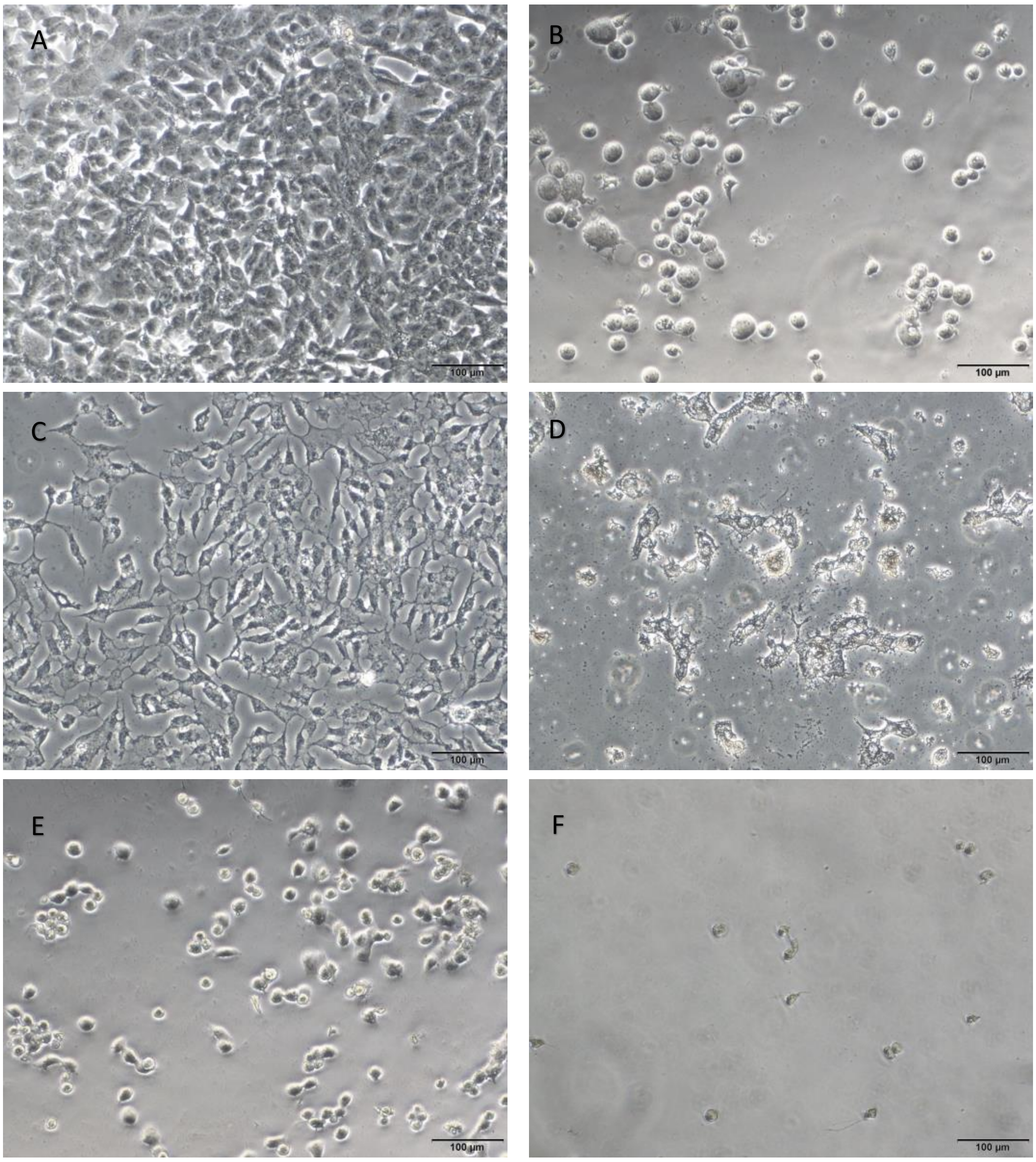

Figure S17. Vero cell culture in control (A), DMSO (B), cisplatin (C), **Ru1** applied (D), **Ru2** applied (E) and **Ir1** applied (F) groups after 48 h of incubation. Scale bars: 100μm

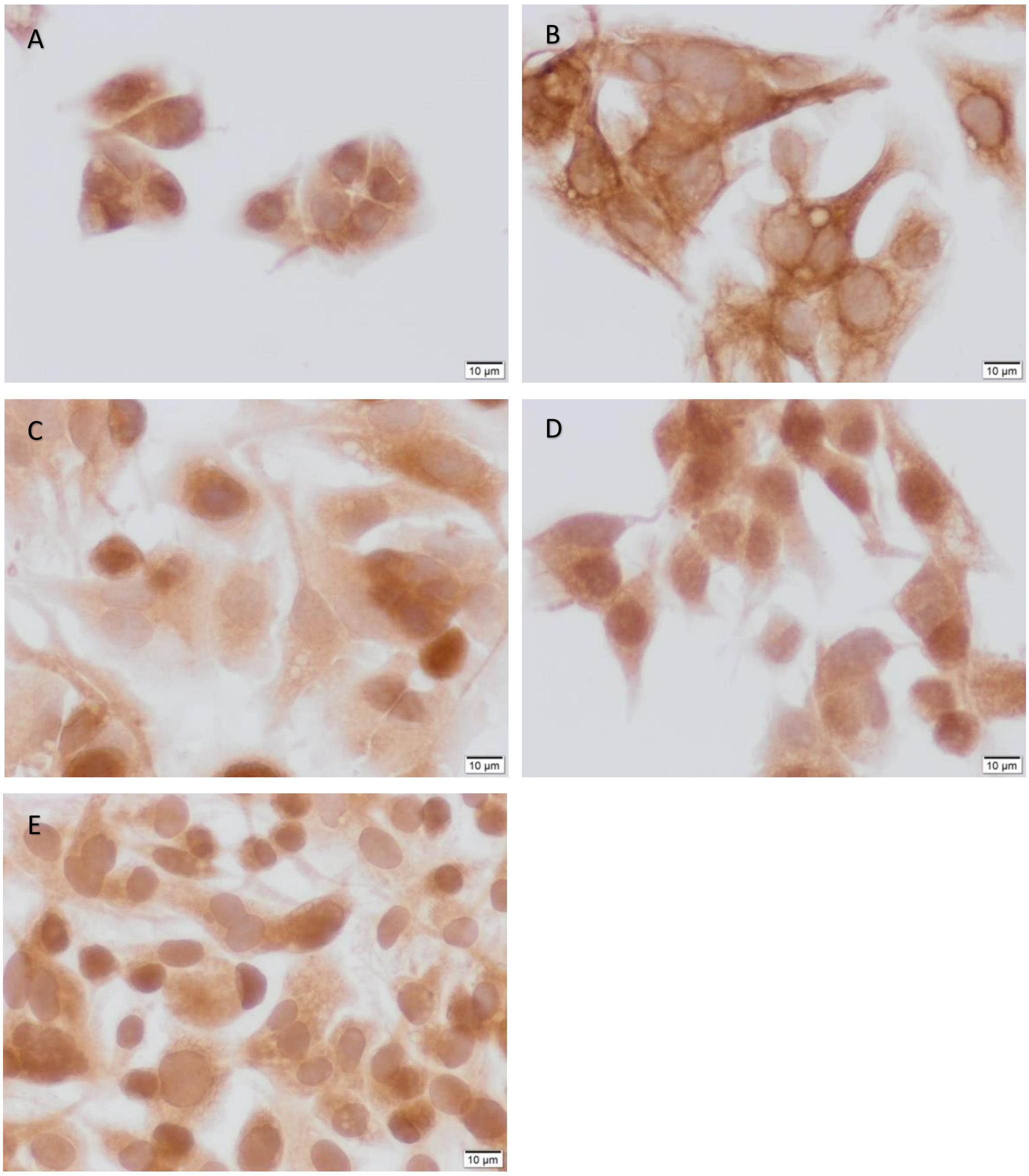

Figure S18. Bax (A), Bcl2 (B) Cas3 (C), RIP3 (D), and RIPK1 (E) immunoreactivities after control administration to HepG2 cells. Scale bars: 10 μm.

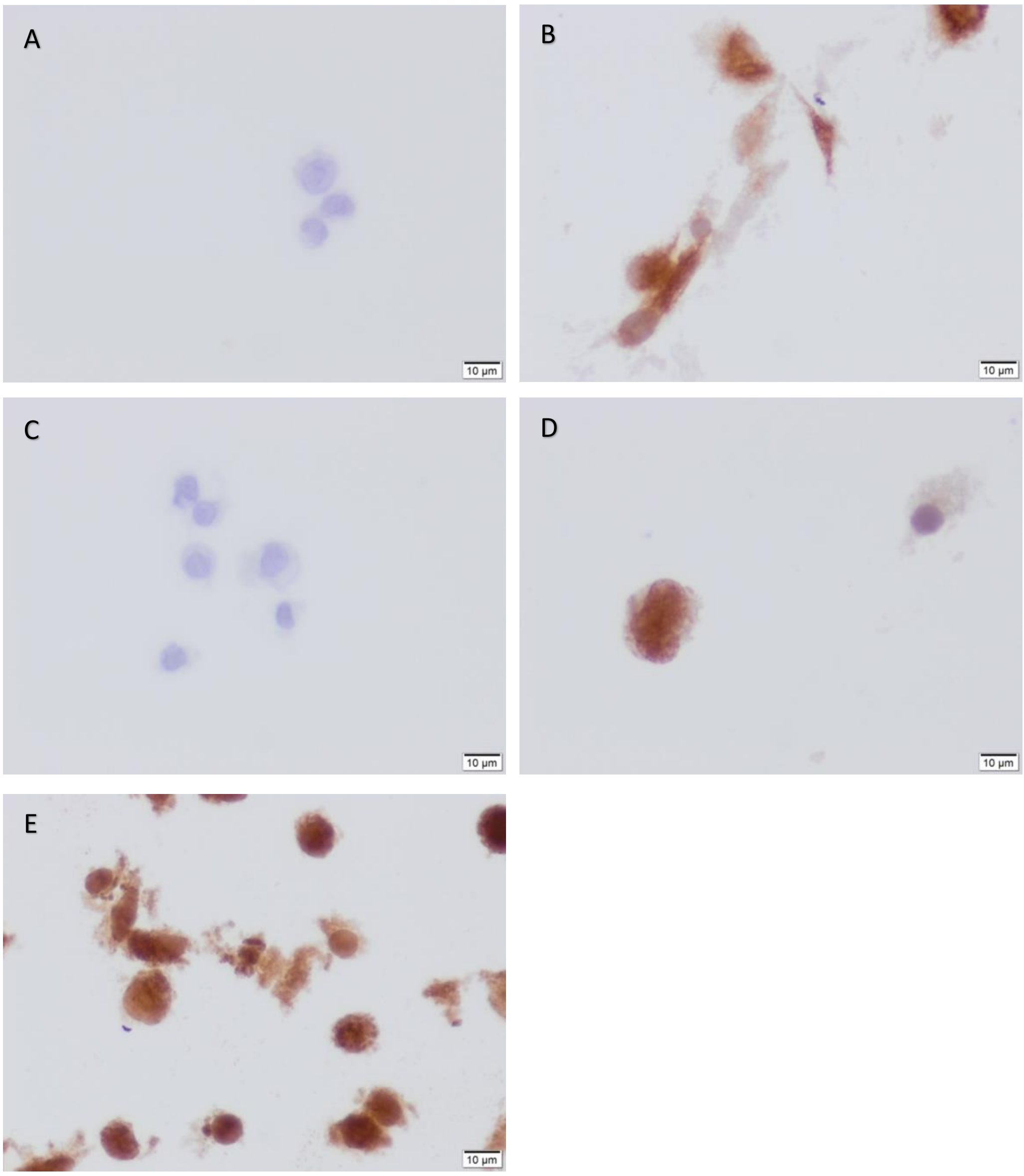

Figure S19. Bax (A), Bcl2 (B) Cas3 (C), RIP3 (D), and RIPK1 (E) immunoreactivities after DMSO administration to HepG2 cells. Scale bars: 10 μm.

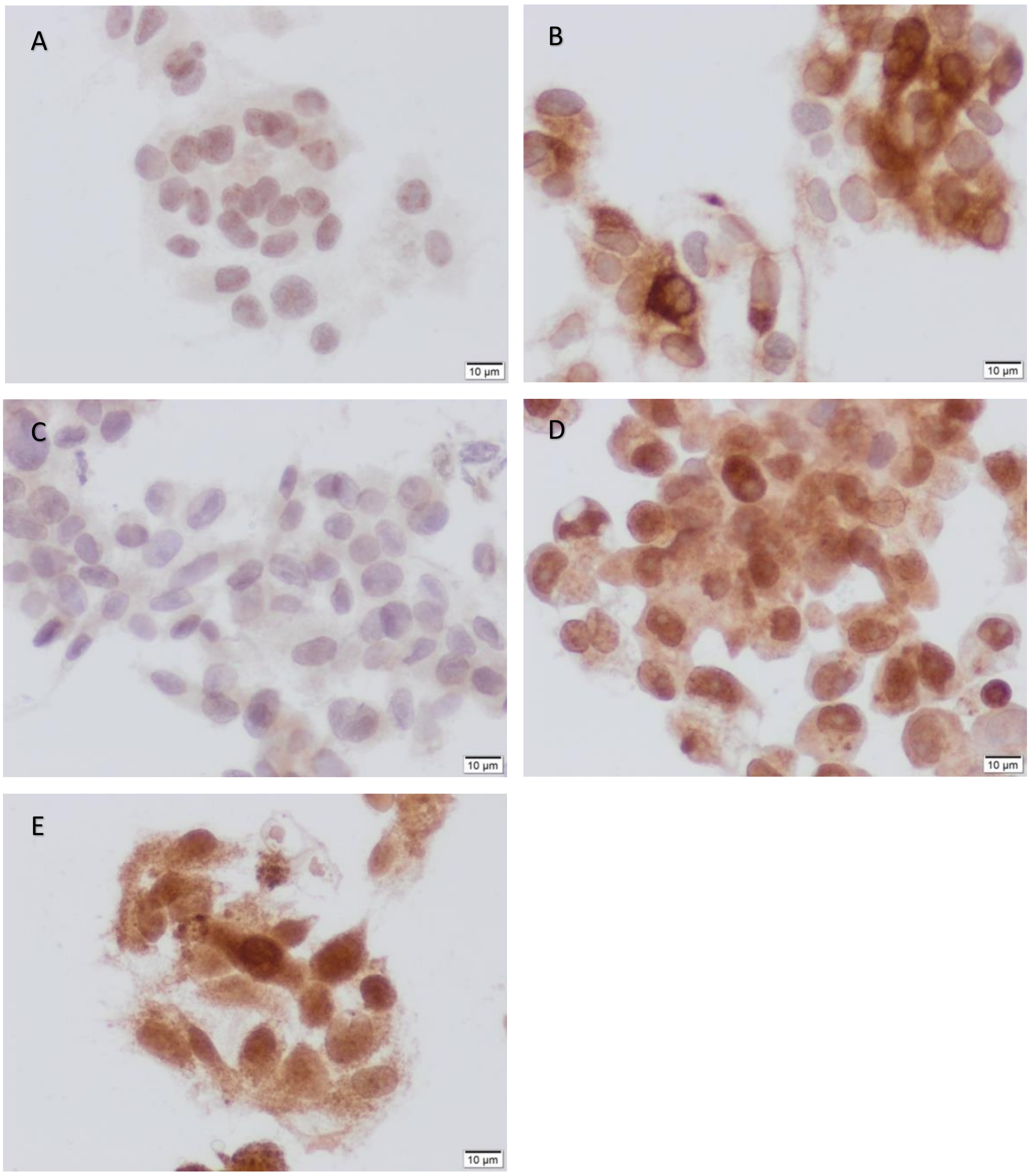

Figure S20. Bax (A), Bcl2 (B) Cas3 (C), RIP3 (D), and RIPK1 (E) immunoreactivities after cisplatin administration to HepG2 cells. Scale bars: 10 μm.

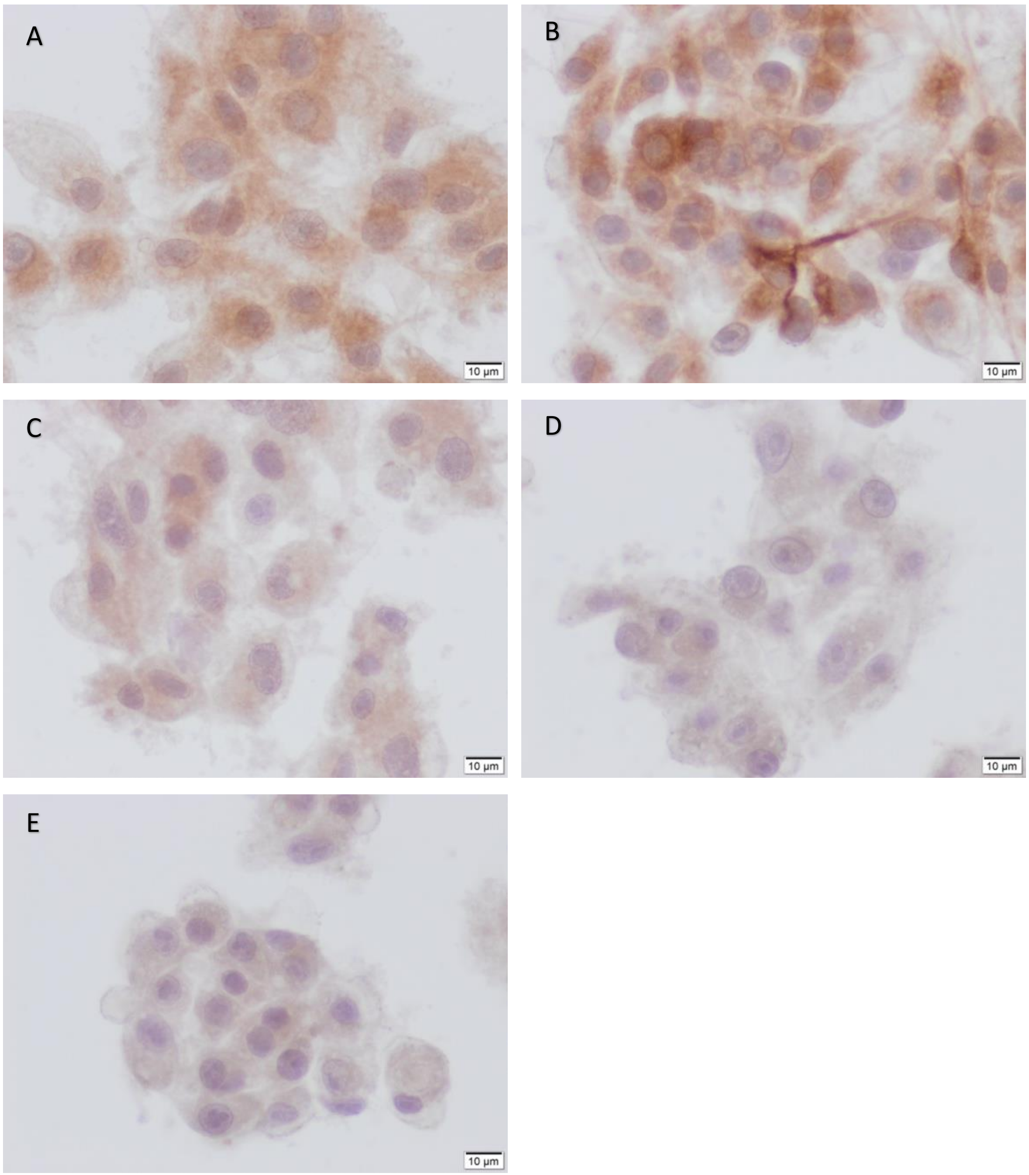

Figure S21. Bax (A), Bcl2 (B) Cas3 (C), RIP3 (D), and RIPK1 (E) immunoreactivities after **Ru1** administration to HepG2 cells. Scale bars: 10 μm.

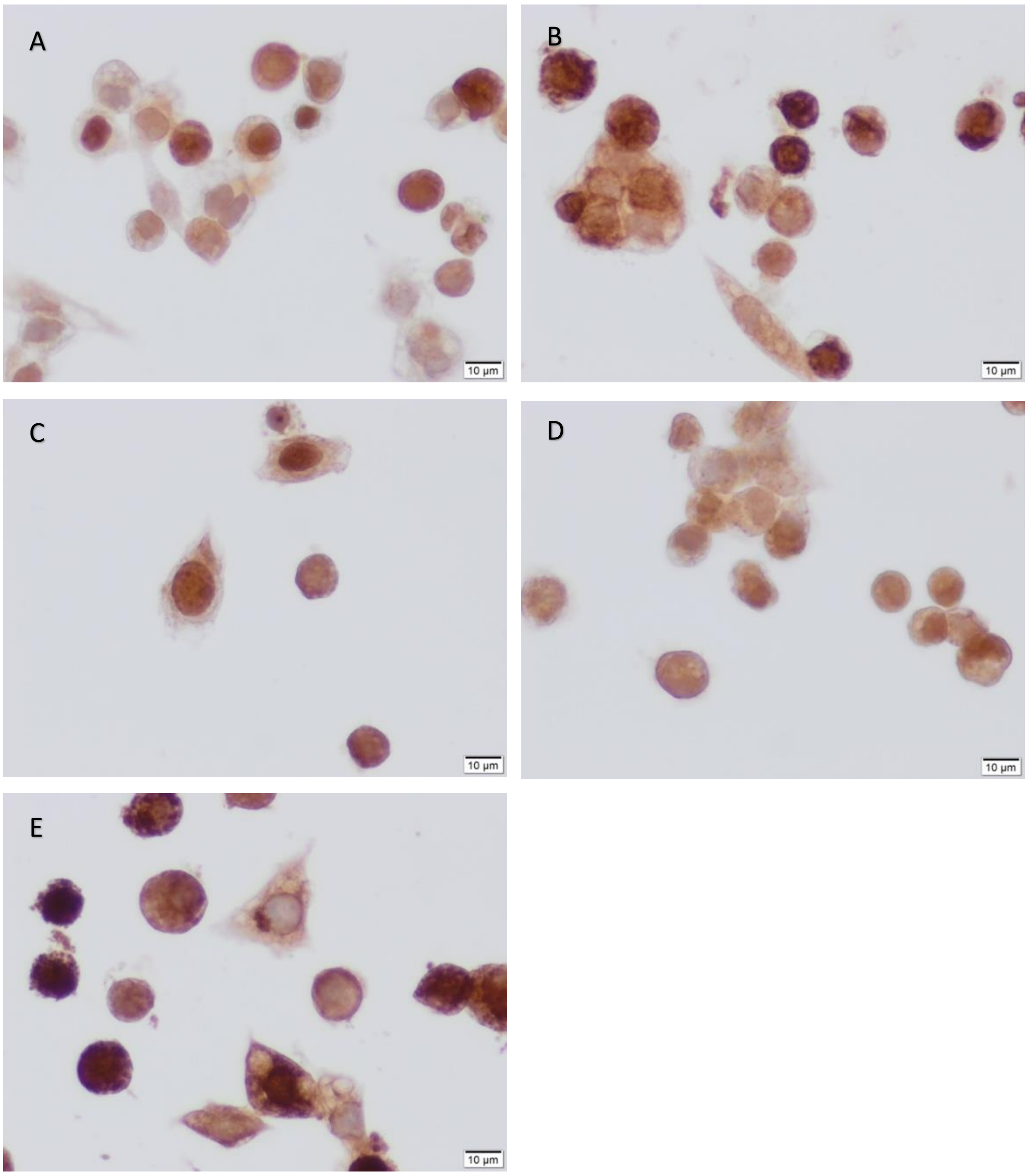

Figure S22. Bax (A), Bcl2 (B) Cas3 (C), RIP3 (D), and RIPK1 (E) immunoreactivities after **Ru2** administration to HepG2 cells. Scale bars: 10 μm.

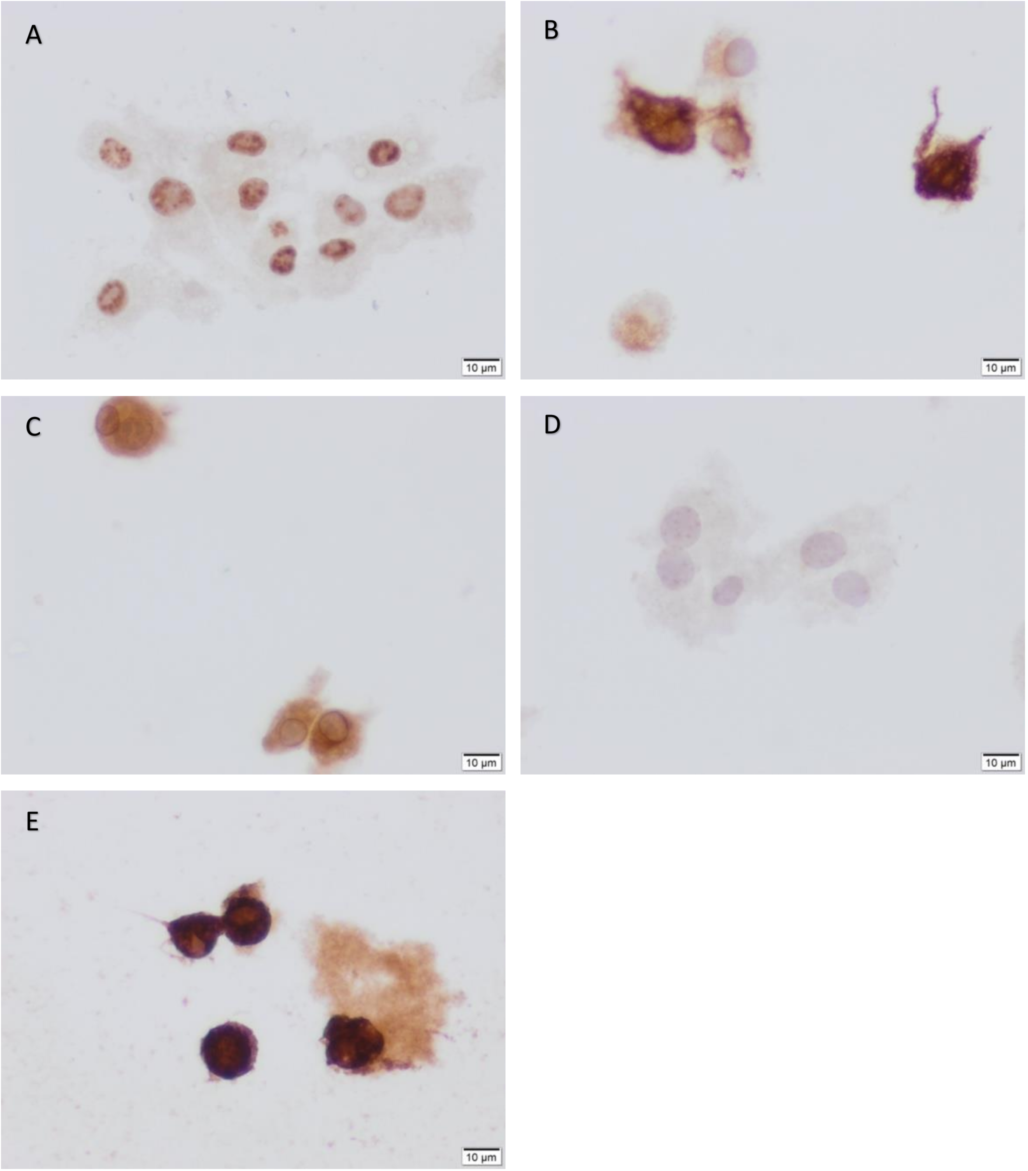

Figure S23. Bax (A), Bcl2 (B) Cas3 (C), RIP3 (D), and RIPK1 (E) immunoreactivities after **Ir1** administration to HepG2 cells. Scale bars: 10 μm.

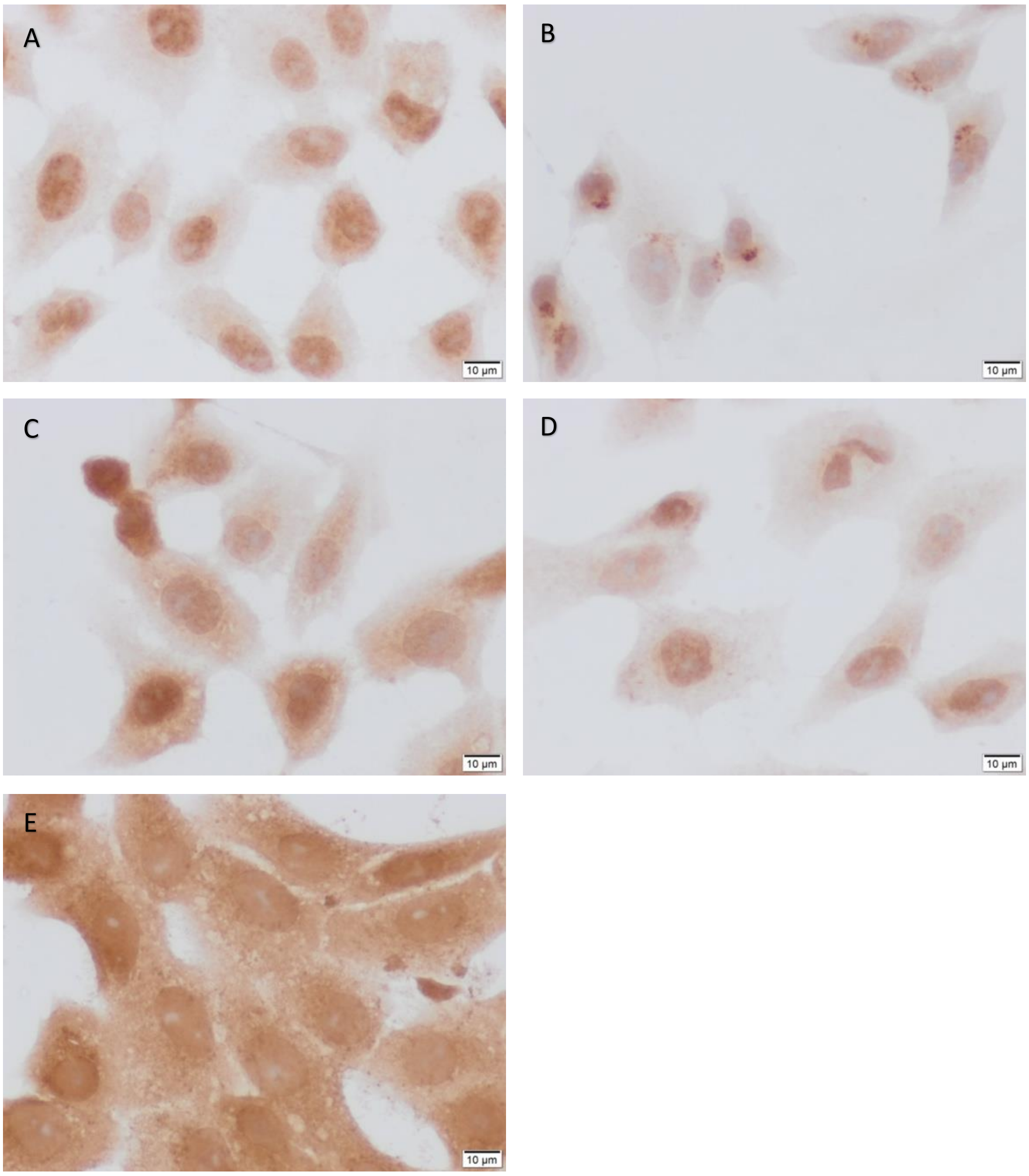

Figure S24. Bax (A), Bcl2 (B) Cas3 (C), RIP3 (D), and RIPK1 (E) immunoreactivities after control administration to Vero cells. Scale bars: 10 µm.

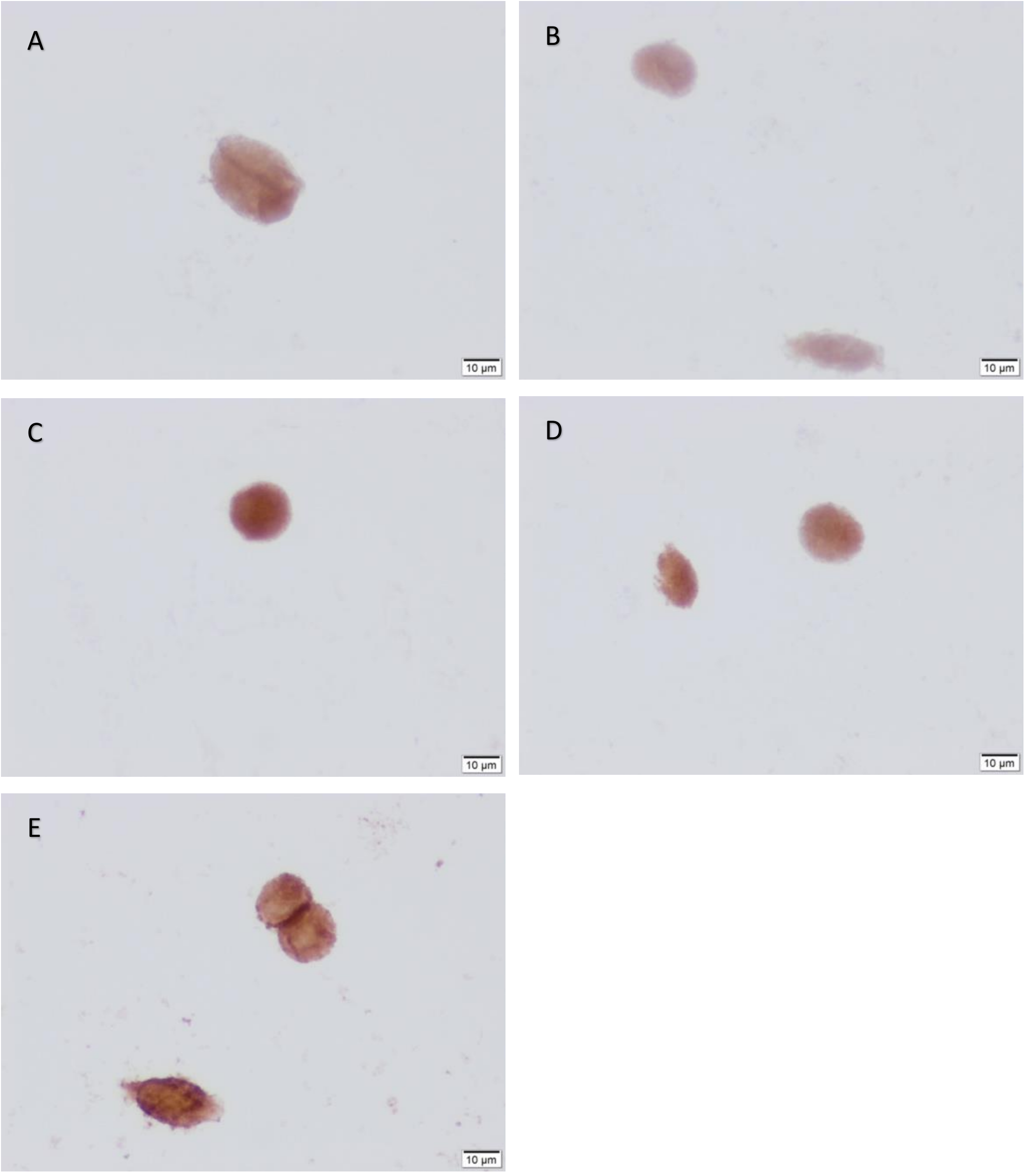

Figure S25. Bax (A), Bcl2 (B) Cas3 (C), RIP3 (D), and RIPK1 (E) immunoreactivities after DMSO administration to Vero cells. Scale bars: 10 μm.

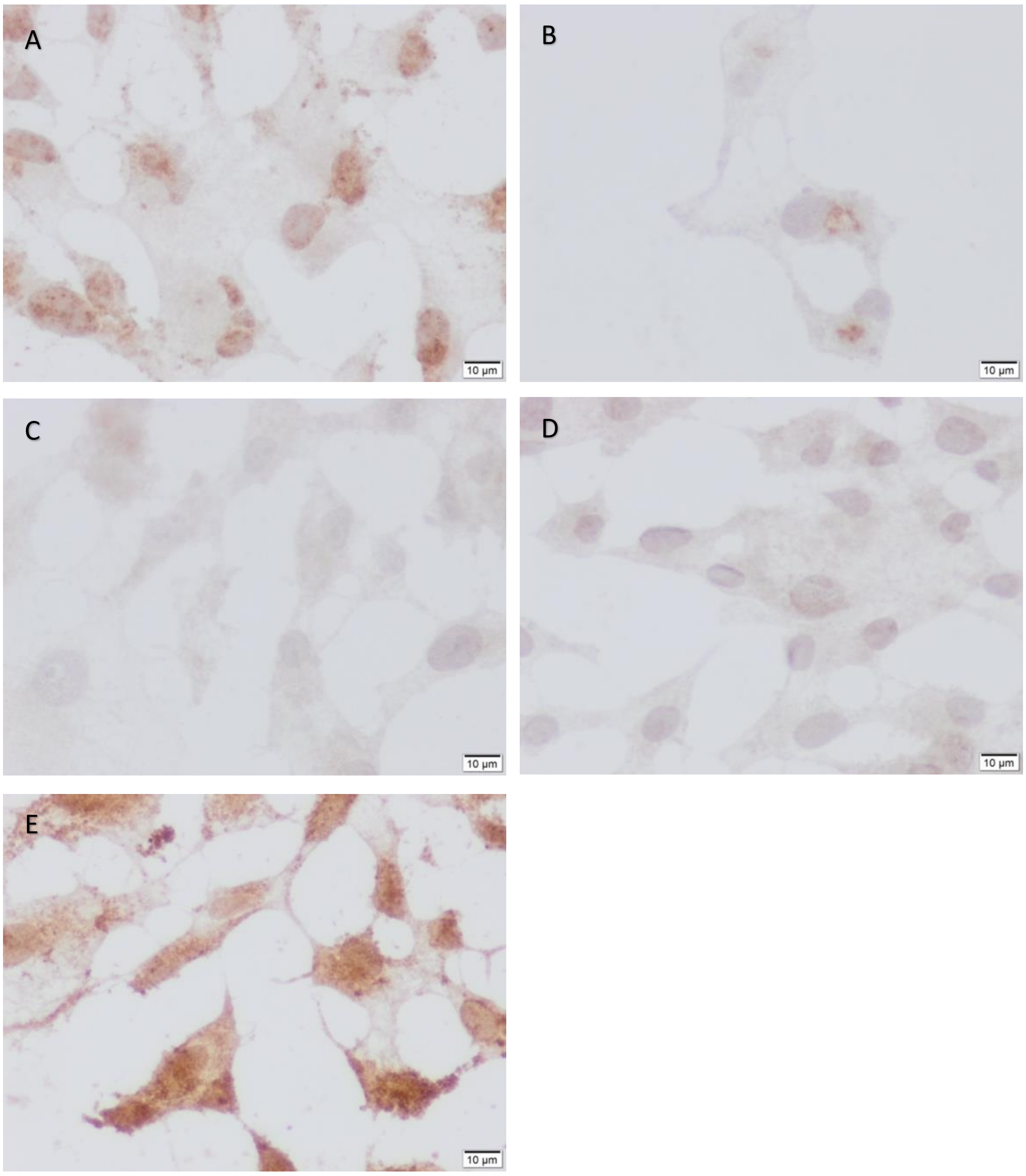

Figure S26. Bax (A), Bcl2 (B) Cas3 (C), RIP3 (D), and RIPK1 (E) immunoreactivities after cisplatin administration to Vero cells. Scale bars: 10 μm.

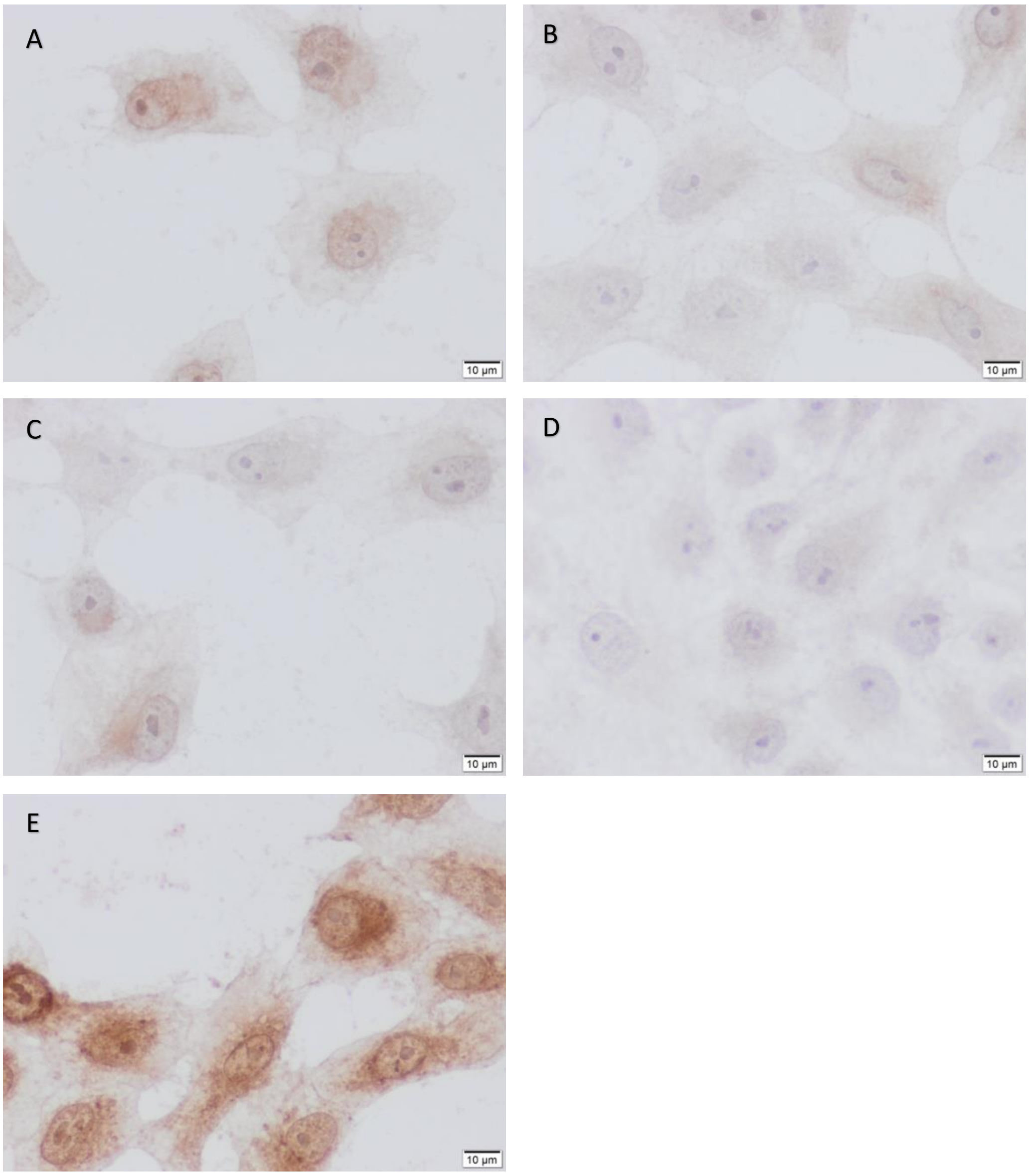

Figure S27. Bax (A), Bcl2 (B) Cas3 (C), RIP3 (D), and RIPK1 (E) immunoreactivities after **Ru1** administration to Vero cells. Scale bars: 10 μm.

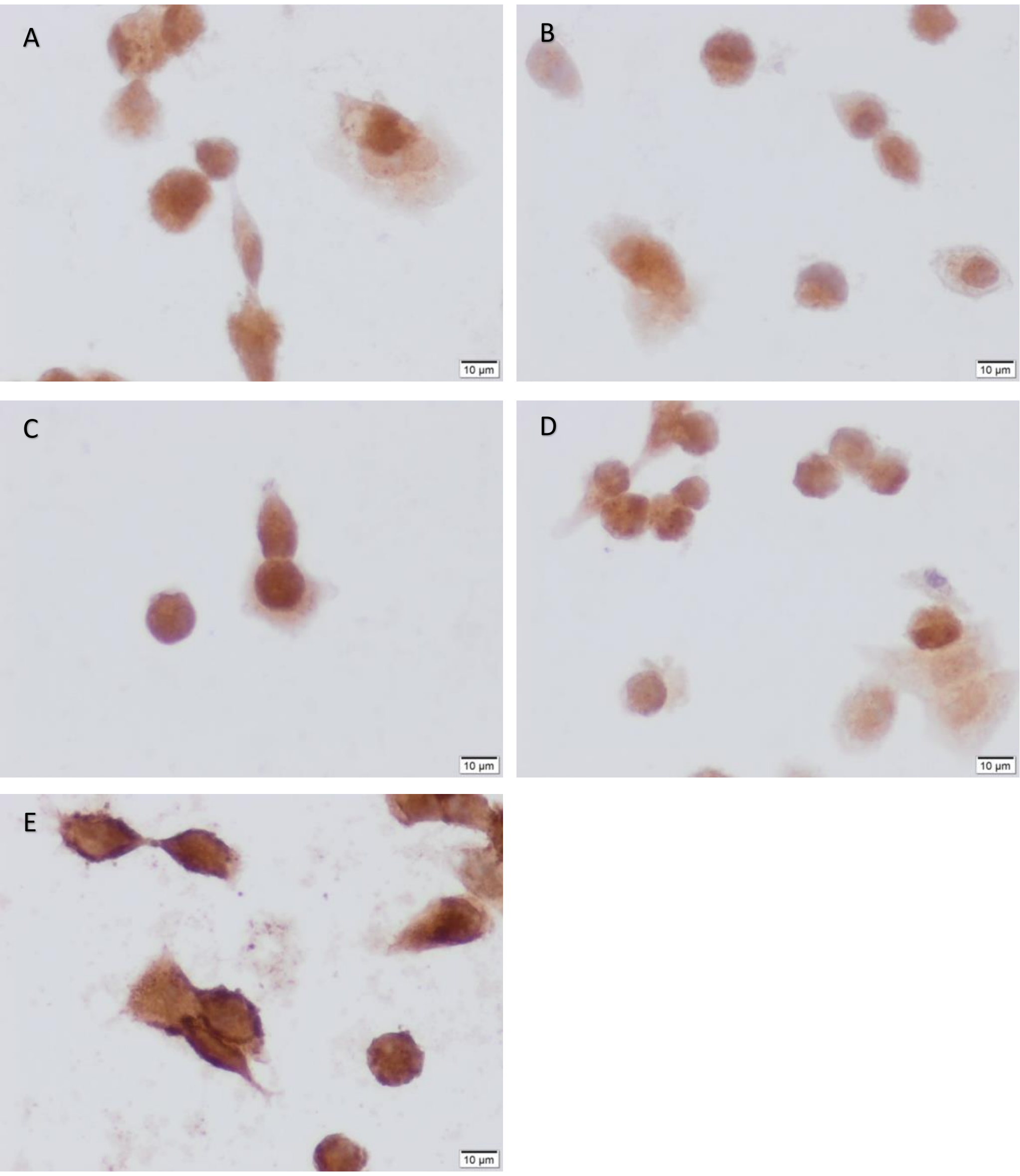

Figure S28. Bax (A), Bcl2 (B) Cas3 (C), RIP3 (D), and RIPK1 (E) immunoreactivities after **Ru2** administration to Vero cells. Scale bars: 10 μm.

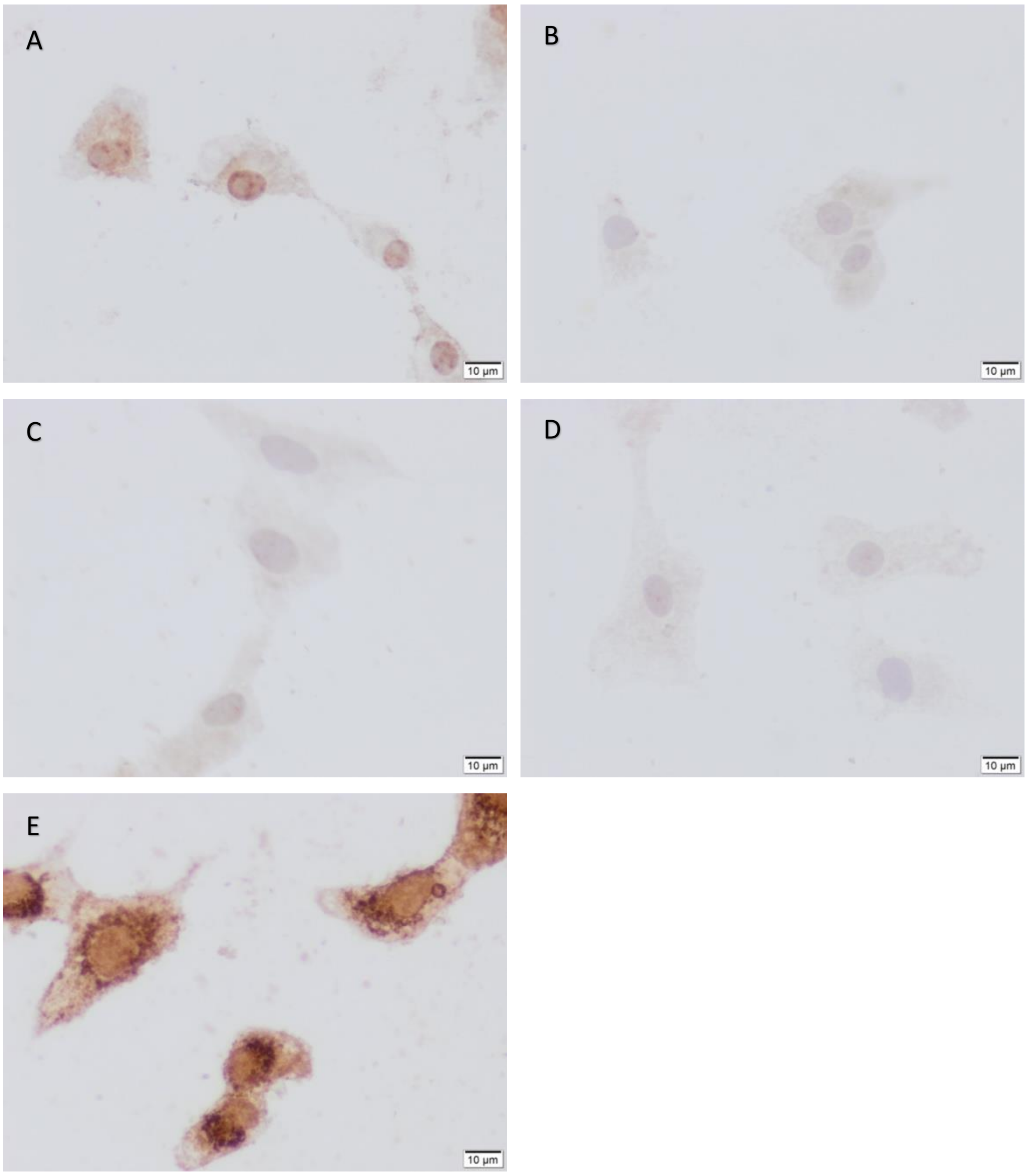

Figure S29. Bax (A), Bcl2 (B) Cas3 (C), RIP3 (D), and RIPK1 (E) immunoreactivities after **Ir1** administration to Vero cells. Scale bars: 10 μm.

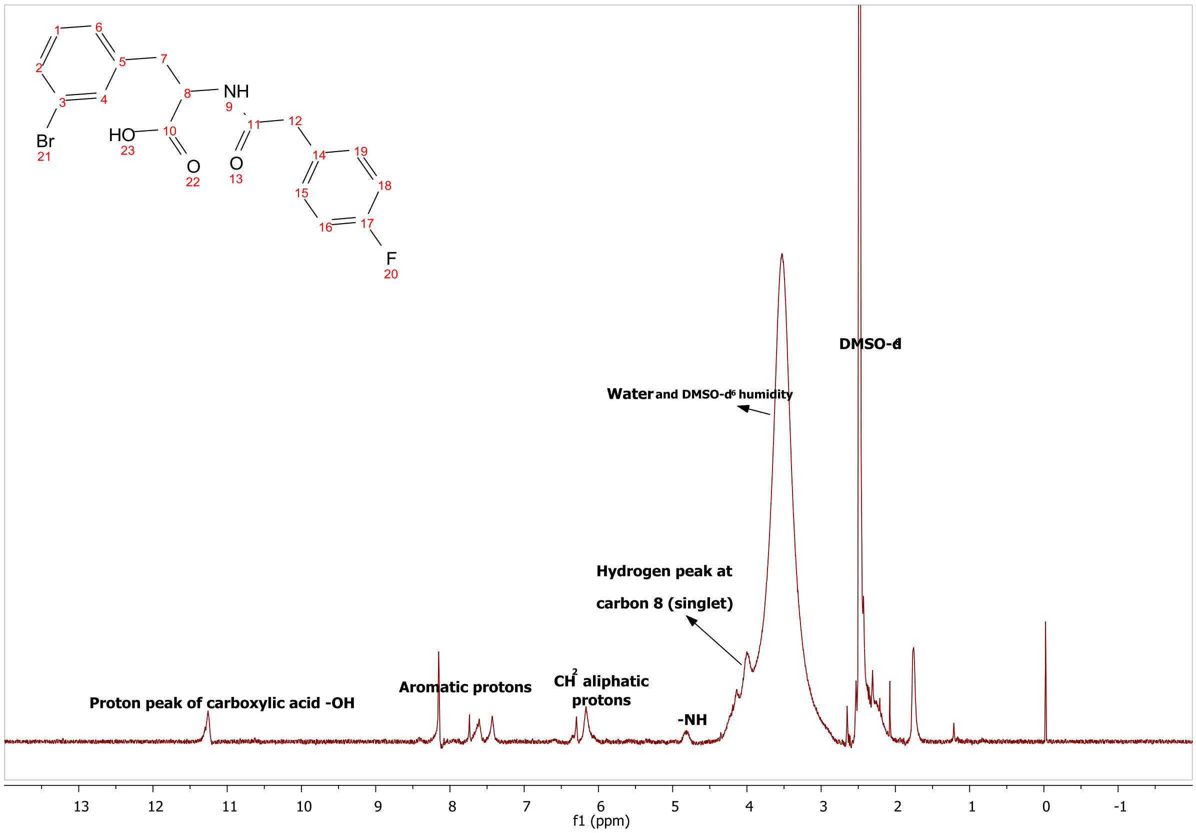

Figure S30. The detailed ^{1}H-NMR spectrum of FS-DNA.

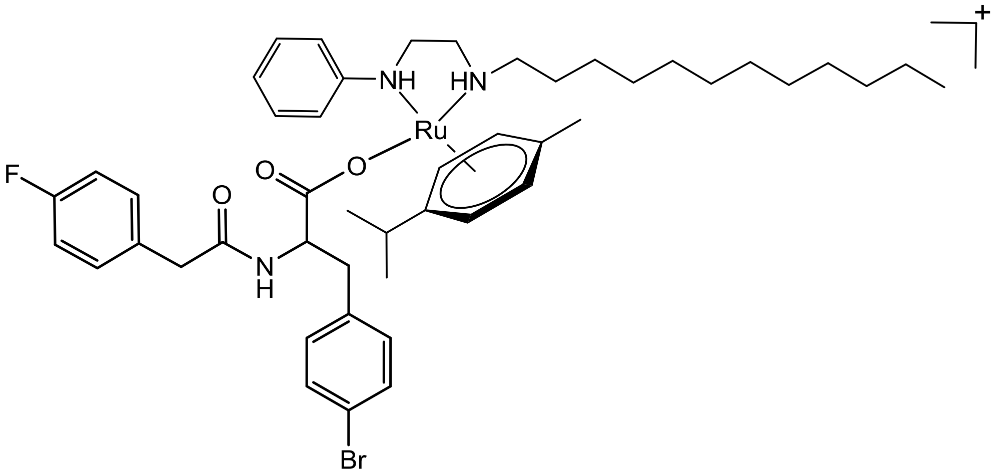

Figure S31. The possible structure resulting from the interaction of FS-DNA with **Ru1**.
